# Exercise physiology and speech pathology support treatment engagement in forensic mental health in community health: evidence from a retrospective cohort study

**DOI:** 10.3389/fpsyt.2026.1964444

**Published:** 2026-10-19

**Authors:** Frances Shawyer, Kate Wilson, Raja Ram Dhungana, Ben Atkinson, Lyndsey Hipgrave, Shrinkhala Dawadi, Joanne Enticott, Graham Meadows

**Affiliations:** 1 Southern Synergy, Department of Psychiatry, School of Clinical Sciences at Monash Health, Faculty of Medicine, Nursing and Health Sciences, Monash University, Melbourne, VIC, Australia; 2 Monash Centre for Health Research and Implementation, Faculty of Medicine, Nursing and Health Sciences, Monash University, Melbourne, VIC, Australia; 3 School of Primary and Allied Health Care, Faculty of Medicine, Nursing and Health Sciences, Monash University, Melbourne, VIC, Australia; 4 Mental Health Program, Monash Health, Dandenong, VIC, Australia

**Keywords:** community health services, exercise physiology, exercise therapy, forensic psychiatry, speech pathology, speech therapy, speech-language pathology, treatment adherence and compliance

## Abstract

**Background:**

Adults completing community-based orders (CBOs) imposed by courts in Victoria, Australia, often present with complex mental health needs and face barriers to treatment engagement. The Forensic Mental Health in Community Health (FMHiCH) program was established to address these challenges through multidisciplinary care within a community health model across multiple sites. At one site, exercise physiology (EP) and speech pathology (SP) were introduced to enhance engagement and support treatment success, however evidence for these interventions in forensic mental health settings is limited. Because the FMHiCH dataset did not contain a direct measure of engagement, this study examined whether EP and/or SP involvement was associated with two behavioural indicators of engagement: treatment completion and treatment goal achievement, and whether greater service dose improved these indicators.

**Methods:**

A retrospective cohort study analysed de-identified FMHiCH data (December 2018–June 2023) from 1,111 care episodes. Mixed-effect logistic regression was used to examine associations between EP and SP involvement and the two treatment outcomes, adjusting for demographic and socio-economic factors. Dose effects were explored using univariate logistic regression for EP and SP contact time and number of contacts.

**Results:**

EP and/or SP involvement was positively associated with both engagement indicators across minimally adjusted (treatment completion: EP OR 5.65 [95% CI 2.41–13.23], SP OR 2.50 [1.18–5.33]; goal achievement: EP OR 6.67 [2.42–18.42], SP OR 4.05 [1.66–9.85]) and fully adjusted models (treatment completion: EP OR 5.68 [2.10–15.37], SP OR 2.44 [0.93–6.36]; goal achievement: EP OR 7.26 [2.32–22.66], SP OR 3.75 [1.38–10.17]). Higher EP and SP contact hours were significantly associated with treatment completion and treatment goal achievement. Demographic factors such as secure housing and employment showed associations with engagement indicators.

**Conclusions:**

Integrating EP and SP into community-based forensic mental health care was associated with improved behavioural indicators of engagement - treatment completion and goal achievement. The findings suggest that these allied health interventions may enhance engagement and rehabilitation for adults completing CBOs. Controlled studies, such as stepped-wedge cluster randomised trials, are warranted to confirm these associations, clarify dose effects and further explore the influence of social determinants of health.

## 1 Introduction

Adults who commit criminal offences in Victoria, Australia, commonly encounter the criminal justice system (CJS) and may receive a range of penalties depending on the nature and seriousness of the offence. Two such penalties are Community Correction Orders (CCOs) and Parole Orders (POs). CCOs allow individuals to remain in the community while completing court-mandated conditions over a specified period. POs apply to individuals transitioning back to the community following imprisonment. Both orders - hereafter referred to as community-based orders (CBOs) - require judicial supervision, stipulating periodic contact with Department of Justice and Community Safety staff to monitor compliance. Breaching the conditions of CBOs may result in further penalties.

Order conditions are often designed to address factors considered contributory to the individual’s offending behaviour. Mental health challenges frequently feature in circumstances surrounding criminal activity (1) and mental health assessment and treatment may therefore be mandated as part of a CBO. Because these orders are completed in the community, and unless specific treatment requirements are stipulated, individuals typically retain greater choice over treatment providers and therapy direction than is possible in custodial settings. Such autonomy is generally considered beneficial for treatment engagement and success (2). However, self-motivation and sustained participation in treatment can be challenging for this population, with treatment disengagement recognised as a significant problem (3). Many adults with mental health conditions completing CBOs also experience co-occurring physical health problems (4, 5), fractured family relationships, unstable housing and other adverse social determinants of health (6, 7) that make it difficult for them to maintain day-to-day stability and complete their CBO satisfactorily. Many of these factors contribute to poor involvement with local health providers such as General Medical Practitioners or other formal health networks and limited health literacy (8, 9), combining to create a lack of the personal resources required to navigate healthcare systems. Rates of recidivism are high, reaching up to 43% in some cohorts (10).

### 1.1 Community mental health services for adults on community-based orders

A range of health and support services have been developed to assist adults completing CBOs (11). However, community-based programs that match an individual’s needs with CJS requirements are difficult to design, implement and sustain. The evidence base for these approaches remains limited, particularly for interventions supporting transition to the community, while broader forensic mental health practice continues to be constrained by gaps in high-quality evidence (12, 13). Additional barriers include resource constraints, workforce shortages, and ongoing training needs among both health and correctional staff (14, 15).

In Victoria, community health services are widely distributed within local neighbourhoods and may provide an accessible setting for the delivery of forensic mental health programs. Their multidisciplinary and holistic approach grounded in an understanding of the social determinants of health may enable them to respond more flexibly and effectively to the diverse needs of people completing CBOs than could be achieved via health services with more rigid operation policies (16). Community health settings may also support therapeutic relationships, which are known to influence successful order completion (17).

As part of Victoria’s Forensic Mental Health Implementation Plan and 10-year reform process, the Forensic Mental Health in Community Health (FMHiCH) program was established across five Victorian sites to provide mental and general health care for adults referred from Community Correctional Services (18). Entry criteria were a) being subject to a CBO, b) diagnosed with a moderate severity mental health disorder, and c) being assessed as presenting a medium-to-high risk of re-offending (18). The program aimed to improve mental and physical health, support compliance with mental health treatment-related order conditions, and facilitate the successful completion of the CBO (18).

Exercise physiology (EP) and speech pathology (SP) services were uniquely included at one of the five sites as part of the multidisciplinary service model. Their inclusion was intended not only to provide direct therapeutic services but also to enhance engagement and trust through interventions that may be perceived as accessible and appealing.

### 1.2 Exercise physiology for adults with mental health needs and a criminal conviction

Exercise Physiologists design, deliver and evaluate exercise interventions for people with acute, sub-acute or chronic health conditions (19). Poor physical health is highly prevalent among people in prison and among those transitioning back into the community, with many experiencing modifiable risk factors for chronic disease, including physical inactivity, smoking, poor nutrition, and problematic alcohol use (9, 20–22). Physical health conditions commonly co-occur with mental health conditions and contribute substantially to overall disease burden and poorer health outcomes (23, 24).

Recognition of the importance of physical health in mental health recovery has led to EPs becoming increasingly integrated within multidisciplinary mental health teams. Outcomes from several studies (25–28) suggest that EPs appear well-equipped and able to generate positive physical and mental health outcomes while also increasing staff awareness of the physical health needs of people receiving mental health care.

### 1.3 Speech pathology for adults with mental health needs and a criminal conviction

Speech Pathologists assess and treat communication and swallowing difficulties. Communication difficulties are disproportionately common among people involved with the justice system and may limit their ability to understand information, communicate effectively, participate in treatment, and engage with rehabilitation processes (29–34). Professional bodies in both Australia and the United Kingdom recognise SPs as important contributors to justice and rehabilitation services (31, 33, 34).

Speech Pathology Australia also identifies SPs as important members of multidisciplinary mental health teams. Despite this recognised role, SP involvement in adult forensic and mental health services in Victoria has traditionally been limited.

### 1.4 Exercise physiology and speech pathology as facilitators of engagement in community mental health care

Beyond their therapeutic roles, the inclusion of EP and SP within FMHiCH reflected broader service objectives related to engagement and participation in care. As described above, many adults completing CBOs experience multiple forms of social disadvantage and associated challenges engaging with health and mental health services. Engagement in forensic mental health settings may be further constrained by low motivation, stigma, distrust, and other structural and relational barriers (35), potentially contributing to a reluctance to engage with traditional health and mental health services.

Consistent with the holistic ethos of community health care, EP and SP were considered disciplines that could provide accessible and meaningful pathways into treatment. EP was conceptualised as a non-stigmatising and “normalising” activity (e.g., gym-based exercise), that may be particularly acceptable to a predominantly male client population and facilitate interpersonal connection in a less confrontational context (20). SP was included not only because of the high prevalence of communication difficulties in justice-involved populations, but also because communication barriers can limit individuals’ ability to understand, participate in, and benefit from treatment and rehabilitation processes (21–23).

Together, these disciplines were expected to support trust-building, reduce barriers to participation, and foster client engagement with the FMHiCH service. In doing so, they aligned with the program’s commitment to holistic care, social inclusion, and improved access for individuals experiencing multiple layers of stigma and disadvantage.

### 1.5 Evidence gap and study rationale

Despite the relevance of both disciplines, a comprehensive literature review conducted in July-August 2022 identified no studies examining EP or SP programs delivered within community-based forensic mental health services (for search details, see Section S1.1.1 and **Supplementary Table S1** in the online supplement). An updated search in September 2025 (Section S1.1.2 and **Supplementary Tables S2** and **S3** in the online supplement) identified only one loosely-related study exploring dance and movement therapy (DMT) with two women serving community correctional orders in Victoria (36). This study provided a theoretical rationale for movement-based approaches but did not evaluate EP, SP, treatment engagement, or forensic mental health care more broadly.

This evidence gap is noteworthy because a central rationale for including EP and SP within the FMHiCH model was their potential to enhance engagement with care. Evaluating whether these disciplines are associated with improved engagement is therefore important for understanding their contribution to the program.

However, the FMHiCH dataset did not include direct measures of engagement such as attendance quality, session participation, or attitudinal indicators. Consistent with the program’s theory of change (37) and with common practice in forensic and mandated-treatment research, where engagement is frequently inferred from observable participation and progression indicators (35, 38), engagement was operationalised using treatment completion and treatment goal achievement. These outcomes reflect sustained involvement in, and progress through, treatment and therefore served as the primary behavioural indicators of engagement in the present study.

### 1.6 Aims

The main aims of this study were to determine whether:

1. Individuals who received EP and/or SP services are more likely than those who did not to complete treatment and achieve their treatment goals.
2. Greater EP and/or SP service dose is associated with treatment completion and achievement of treatment goals.

A secondary aim was to explore associations between treatment outcomes and demographic and contextual variables to provide additional context to the findings.

### 1.7 Hypotheses

It is hypothesised that treatment success, defined as treatment completion and/or treatment goal achievement, would be positively associated with:

- Involvement of EP and/or SP services; and
- Greater EP and SP service dose, measured through clinician-reported contacts logged to individual data profiles.

## 2 Methods

### 2.1 Setting and study population

We conducted a retrospective cohort study using cross-sectional FMHiCH health service data from the period December 2018 through to June 2023. The de-identified dataset included all episodes of care over this time (1111 cases). Service users were adults meeting the entry criteria as described in the Introduction.

### 2.2 Interventions - exercise physiology and speech pathology services

Upon entry to the FMHiCH program, a Key Worker conducts an intake assessment to identify appropriate services (at least two for eligibility), including potentially EP and SP. Based on factors such as readiness to engage, referral may occur at intake or at any point thereafter, initiated by any team member or requested by the consumer.

For EP services, the EP conducts a comprehensive assessment of the individual’s physical health, identifying any conditions or injuries that may be impacted by exercise, and collaborating with the consumer to establish personalised goals (e.g., strength building). Sessions are usually held weekly, though frequency may vary depending on consumer needs and preferences, with an emphasis on fostering independence and facilitating community-based exercise engagement. The duration of EP input is tailored to each individual and may span weeks to months, concluding when the consumer’s legal order ends.

For SP services, the SP contacts the individual to arrange an initial appointment, usually in person or online. The first session is exploratory, focussing on communication needs and goals, often shaped by the client and external stakeholders (e.g., Corrections Victoria or the court). Dysphagia is rarely a concern in this setting. The service is highly flexible and client-led, with no rigid discharge criteria. Intervention includes formal and informal communication assessments, development of a communication profile, and goal-oriented therapy. Common targets include functional tasks (e.g., ordering coffee) and interpersonal communication (e.g., managing conflict). Support continues as needed within the timeframe of the individual’s forensic order. The role is guided by clinical autonomy and aligns with the principles outlined in the 2022 Speech Pathology Scope of Practice in Australia.

### 2.3 Variables

Variables for analysis included demographic, clinical (e.g., International Classification of Diseases, 10th Revision [ICD-10] mental health conditions), service use (e.g., contacts with specific professional disciplines), and treatment outcomes (e.g., treatment completion).

Outcome variables were entered by the client’s key worker. As noted earlier, the FMHiCH database did not contain a direct measure of treatment engagement; therefore, engagement was operationalised using the two outcome variables - treatment completion and treatment goal achievement - as behavioural indicators. The categorisation of both outcome fields was conducted in consultation with FMHiCH staff.

The first outcome variable*, treatment completion*, was derived from a “reason for discharge” field. Responses were dichotomised as *completed treatment* (coded as 1) or *did not engage in treatment* (coded as 0). Cases in which the response did not pertain to treatment engagement - such as becoming unavailable (e.g., “moved out of area”) or indicating that no treatment was required - were excluded from the regression analysis. Further details are provided in **Supplementary Table S4** in the online supplement.

The second outcome variable, *treatment goal achievement* (added to the database in September 2020), was also dichotomised: *treatment goal achieved* and *treatment goal partially achieved* were coded as 1, and *treatment goal not achieved* was coded as 0. Other non-relevant responses were excluded. Partial achievement was combined with full achievement in the categorisation because there was no objective or standardised distinction between partial and full achievement, and partial achievement was generally regarded by clinicians as meaningful progress towards individualised treatment goals. Combining these categories reduced reliance on a distinction that was subject to clinician judgement and known variability in operational application. Further details are provided in **Supplementary Table S5** in the online supplement.

Postcode data was included based on the understanding of socio-economic status as being likely to be an important factor in understanding outcomes and a potential confounder. For instance, increased rates of mental disorders and psychological distress are associated with increased socioeconomic disadvantage of area (39). Social circumstances come with social capital which may influence health and criminal outcomes for affected individuals. Proximity to the service is also a factor since there are likely to be higher referral thresholds for people living further away and lower levels of outreach – meaning they may be referred at a more severe level of illness compared to those living nearby. Proximity is also likely to influence treatment engagement and completion. We examined the Index of Relative Socio-Economic Disadvantage (IRSD) for areas based on the postcode information. As one of the Socioeconomic Indexes for Areas (SEIFA) produced by the Australian Bureau of Statistics, it is a composite index based on census information regarding socio-economic factors and resources within an area, including weighted contributions from income, education, employment, type of housing and other variables. Proximity to the service was measured in kilometres as straight distance (as the crow flies), land transport distance, and travel time in minutes using https://www.freemaptools.com/distance-between-australia-postcodes.htm.

### 2.4 Statistical analysis

Stata version 18 (StataCorp LLC, College Station, TX, USA) was employed to analyse the data. We used client-level data for descriptive statistics to accurately represent the population served and avoid duplication. Categorical variables were summarised using cross tabulations to present frequencies and proportions across study groups. Differences in categorical distributions between groups were evaluated using the Pearson chi square test, or Fisher’s exact test when expected cell counts were fewer than five. For chi-square analyses, the strength of association was quantified using Cramer’s V. Effect sizes were reported for all inferential analyses. For chi-square tests, Cramer’s V was interpreted according to Cohen’s (40) conventions (small = 0.10, medium = 0.30, large = 0.50). For logistic regression analyses, odds ratios (ORs) and 95% confidence intervals (CIs) were reported.

For regression analyses, we used episode-level data because outcomes and service characteristics are defined per episode, and this approach preserves variability and statistical power. Here, models accounted for clustering of episodes within clients to ensure valid inference. Continuous variables including travel time, crow distance, and land distance were categorised into lower, middle, and upper groups for descriptive analyses using the Stata cut() function, which creates grouped categories based on the range of the variable values. These variables were retained as continuous variables in regression models.

For the first aim, mixed-effect logistic regression analysis was employed to construct both univariate and multivariable regression models. These models aimed to discern whether the receipt of EP and/or SP services was associated with treatment completion and achievement of treatment goals. The minimally adjusted model (model 1) included age, sex, country of birth, and IRSD decile scores, while fully adjusted models (model 2) further incorporated covariates such as marital status, language, Aboriginal status, employment, living arrangement, health conditions, and crow distance. Covariates were selected using a combination of statistical criteria (univariate association of *p* < 0.20) and *a priori* substantive considerations, consistent with recommendations that model development should be informed by both statistical evidence and subject-matter knowledge (41). Demographic, socioeconomic, health, and service-access factors were considered as potential confounders of the relationship between receipt of EP and/or SP services and treatment outcomes, informed by prior literature (e.g., 4, 5-9) and clinical knowledge.

In a sensitivity analysis, the Multiple Imputation by Chained Equations (MICE) model was employed to impute missing values in outcome variables and other covariates, including IRSD decile, crow distance, employment status, and health conditions. This process generated 10 imputed datasets (seed of 54321, 100 burn-ins). All variables present in the fully adjusted model were considered for imputation.

For the second aim, univariate logistic regression was used to evaluate the impact of dosage – measured by contact time and number of contacts – of EP and SP services on treatment success. Data collected on the number of contacts distinguished between direct and indirect interactions, with only direct contacts used in the analysis. Direct contacts entailed direct communication between professionals and clients, while indirect contacts involved interactions related to clients but not involving direct engagement with them (e.g., making a phone call pertaining to the client). EP direct contact time comprised pre-exercise discussion and assessment together with exercise delivery, the latter constituting the majority of direct contact time. For contact time, the primary predictor was the natural logarithm of the time receiving EP or SP per episode in minutes by the clients. Because a few clients (*n* = 2) had more than one observation, and responses from the same individual may be correlated, the model used cluster-robust standard errors at the client level to account for within-person dependence. This approach provides valid inference without requiring estimation of a random effects structure, which was unsuitable due to the small number of observations per client.

Results are presented in three stages to guide interpretation of the main analyses. First, we describe overall client characteristics and examine their associations with treatment outcomes to provide context for the sample. Second, we summarise patterns of participation in EP and SP, including differences in client characteristics by participation status. Third, we present the main regression analyses examining associations between EP and SP involvement and treatment outcomes (Aim 1), followed by dose-response analyses (Aim 2).

### 2.5 Research and ethics approval

The project evaluation proposal was reviewed by the Monash Health Human Research Office (Research Support Services) who advised that, as a Quality Assurance project, it was exempt from Human Research Ethics Committee (HREC) review and could be approved through an alternate pathway. Approval and authorisation to commence was received on 19 April 2023 (Monash Health Reference Number: RES-23-0000-247Q). The project also was registered with the Monash University Human Research Ethics Committee on 25 October 2023 (Monash University Project Number: 40788).

## 3 Results

Between December 5, 2018, and July 4, 2023, 1,331 clients were admitted to the program. Of these, 220 had no recorded discharge date, suggesting they were still receiving services, and were excluded. The remaining 1,111 unique individuals (counted once regardless of multiple episodes) were included in the descriptive analysis.

Treatment engagement was operationalised using two behavioural indicators: treatment completion and treatment goal achievement. Results are presented in three stages. First, overall client characteristics are described. Second, patterns of participation in EP and SP are summarised. Third, we present the main regression analyses examining associations between EP and SP involvement and treatment outcomes (Aim 1), followed by dose-response analyses (Aim 2). The descriptive and comparative analyses are presented to provide a more comprehensive understanding of the client population and service use patterns within the program.

### 3.1 Sample characteristics and contextual analyses

#### 3.1.1 Overall client characteristics

**Table 1** provides the overall sample characteristics. Clients of the service were mostly born in Australia (81%), aged between 26–40 years (54%), male (80%), single (68%), unemployed (75%). Nearly all clients spoke English (97%), and the majority had secure housing (76%).

**Table 1:** Sample characteristics.

Variable	Available *n*	Results *n* (%)
Country of birth	1097	
Australia		886 (80.8)
Asia		87 (7.9)
Oceania		47 (4.3)
North America		3 (0.3)
Europe		33 (3.0)
South America		4 (0.4)
Africa		37 (3.4)
Age category	1110	
18-25		236 (21.3)
26-40		597 (53.8)
41 and above		277 (25.0)
Sex	1111	
Female		220 (19.8)
Male		891 (80.2)
Marital status	1111	
De facto		124 (11.2)
Divorced		29 (2.6)
Married		63 (5.7)
Not known		104 (9.4)
Separated		39 (3.5)
Single		752 (67.7)
Preferred language	1109	
English		1075 (96.9)
Others		34 (3.1)
Aboriginal status	1111	
Not Indigenous		1029 (92.6)
Aboriginal or Torres Strait Islander		65 (5.9)
Declined to answer		17 (1.5)
Employment status	770	
Unemployed		577 (74.9)
Employed		176 (22.9)
Other occupation		17 (2.2)
Refugee status	1039	
Not a refugee or asylum seeker		1033 (99.4)
Refugee or asylum seeker		6 (0.6)
Carer status	73	
Does not have a carer		57 (78.1)
Has a carer		16 (21.9)
Living arrangement	1111	
Lives alone		87 (7.8)
Lives with family		414 (37.3)
Lives with others		224 (20.2)
Not stated		386 (34.7)
Accommodation type	555	
Homeless		46 (8.3)
Emergency/transitional housing		15 (2.7)
Secure housing		423 (76.2)
Other		71 (12.8)
Need for interpreter services	1108	
Interpreter needed		14 (1.3)
No interpreter needed		1094 (98.7)
Health conditions	833	
Mental illness		364 (43.7)
Alcohol and other drugs		426 (51.1)
Physical illness or injury		36 (4.3)
Others		7 (0.8)
ICD-10 Mental health conditions^1^	505	
Organic disorders (F00 – F09)		1 (0.2)
Substance use disorders (F10 - F19)		15 (3.0)
Schizophrenia spectrum disorders (F20 - F29)		36 (7.1)
Mood disorders (F30 - F39)		183 (36.2)
Anxiety and stress-related disorders (F40 - F49)		219 (43.3)
Behavioural syndromes (F50 - F59)		1 (0.2)
Personality disorders (F60 - F69)		16 (3.2)
Intellectual disability (F70 - F79)		1 (0.2)
Developmental disorders (F80 - F89)		3 (0.6)
Behavioural/emotional disorders (F90 - F98)		18 (3.6)
Unspecified mental disorder (F99)		12 (2.4)
IRSD^2^	1087	
< 50^th^ percentile		495 (45.5)
≥ 50^th^ percentile		592 (54.5)
Land distance category	1084	
< 6 kms		329 (30.4)
6–10 kms		388 (35.7)
≥ 11		367 (33.9)
Travel time (minutes)	1084	
< 8 mins		304 (28.0)
8–14 mins		413 (38.1)
≥ 15 mins		367 (33.9)

^1^ The listed codes correspond to the groupings of mental and behavioural disorders as outlined in the International Classification of Diseases, 10th revision (ICD-10). They are as follows:

F00-F09 Organic, including symptomatic, mental disorders

F10-F19 Mental and behavioural disorders due to psychoactive substance use

F20-F29 Schizophrenia, schizotypal and delusional disorders

F30-F39 Mood [affective] disorders

F40-F49 Neurotic, stress-related and somatoform disorders

F50-F59 Behavioural syndromes associated with physiological disturbances and physical factors

F60-F69 Disorders of adult personality and behaviour

F70-F79 Mental retardation

F80-F89 Disorders of psychological development

F90-F98 Behavioural and emotional disorders with onset usually occurring in childhood and adolescence

F99 Unspecified mental disorder

^2^ IRSD, Index of Relative Socio-economic Disadvantage.

#### 3.1.2 Associations with treatment outcomes

Associations between client characteristics and the two treatment outcomes are presented in **Supplementary Tables S6** (chi-square analysis using client-level data) and **Supplementary Table S7** (odds ratios using episode-level data), and by treatment goal achievement in **Supplementary Table S8** (chi-square) and **Supplementary Table S9** (odds ratios) (online supplement).

For treatment completion, a higher likelihood of completion was observed among clients born in Asia, those aged ≥41 years, those in a de facto relationship or married, and those who were employed. In contrast, homelessness, Aboriginal or Torres Strait Islander status, and certain mental health conditions - specifically schizophrenia spectrum disorders and behavioural/emotional disorders with onset in childhood - were associated with a lower likelihood of completion. Greater travel time was also associated with higher likelihood of completion, and distance measures showed a similar directional pattern, although not all categories reached statistical significance. For treatment goal achievement, fewer variables reached statistical significance. Employment was associated with a higher likelihood of achieving treatment goals, while homelessness and schizophrenia spectrum disorders were associated with a lower likelihood. Other variables, including age, were not statistically significant predictors in this analysis, although the direction of several associations was consistent with those observed for treatment completion.

#### 3.1.3 Participation in exercise physiology and speech pathology

The referral rate for EP was 11.7% (n = 130) and for SP it was 7.1% (n = 79). Distributions of treatment outcomes by EP and SP participation are presented in **Supplementary Tables S10** and **S11**, and client characteristics stratified by participation status are presented in **Supplementary Tables S12**–**S15**.

Participation in EP was more likely among clients in de facto relationship or who were divorced compared to those who were single.

Participation in SP was associated with age, employment status and health conditions. Clients aged ≥41 years showed marginally higher likelihood of participation. Individuals in “other” occupational categories (including home duties, students, and other roles) were more likely to participate compared to those who were unemployed, while individuals with alcohol and other drug-related conditions were less likely to participate than those with a primary mental health condition.

### 3.2 Main findings

Having characterised the sample and patterns of participation in EP and SP, we next examined whether these interventions were associated with treatment outcomes.

#### 3.2.1 Associations between exercise physiology, speech pathology and treatment outcomes (Aim 1)

For the treatment completion variable, data were available for 619 clients (611 with complete data and 8 with missing data) corresponding to 732 service episodes (723 complete, 9 missing). For treatment goal achieved, data were available for 754 clients (557 complete, 197 missing), corresponding to 889 service episodes (660 complete, 229 missing).

In the final analytical models, all 732 episodes for treatment completion and 889 episodes for treatment goal achievement had outcome and intervention data. Of these, 723 and 660 episodes, respectively, had complete information. Missing values were addressed using multiple imputation. Accordingly, complete case analyses included 723 episodes for treatment completion and 660 episodes for treatment goal achievement. **Figures 1** and **2** present the corresponding flow diagrams.

**Figure 1:**
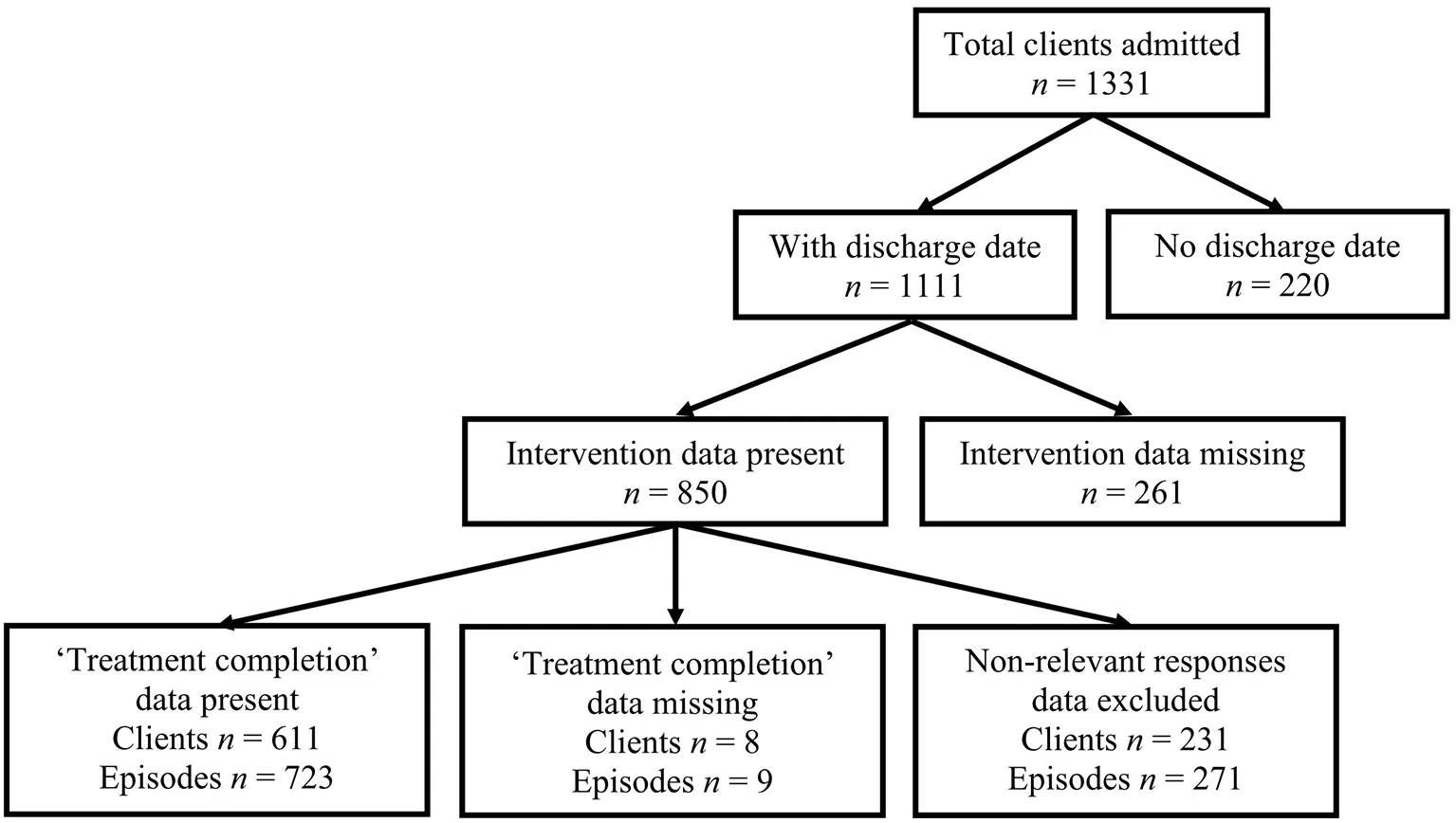
Flowchart of sample derivation for the ‘treatment completion’ analysis.

**Figure 2:**
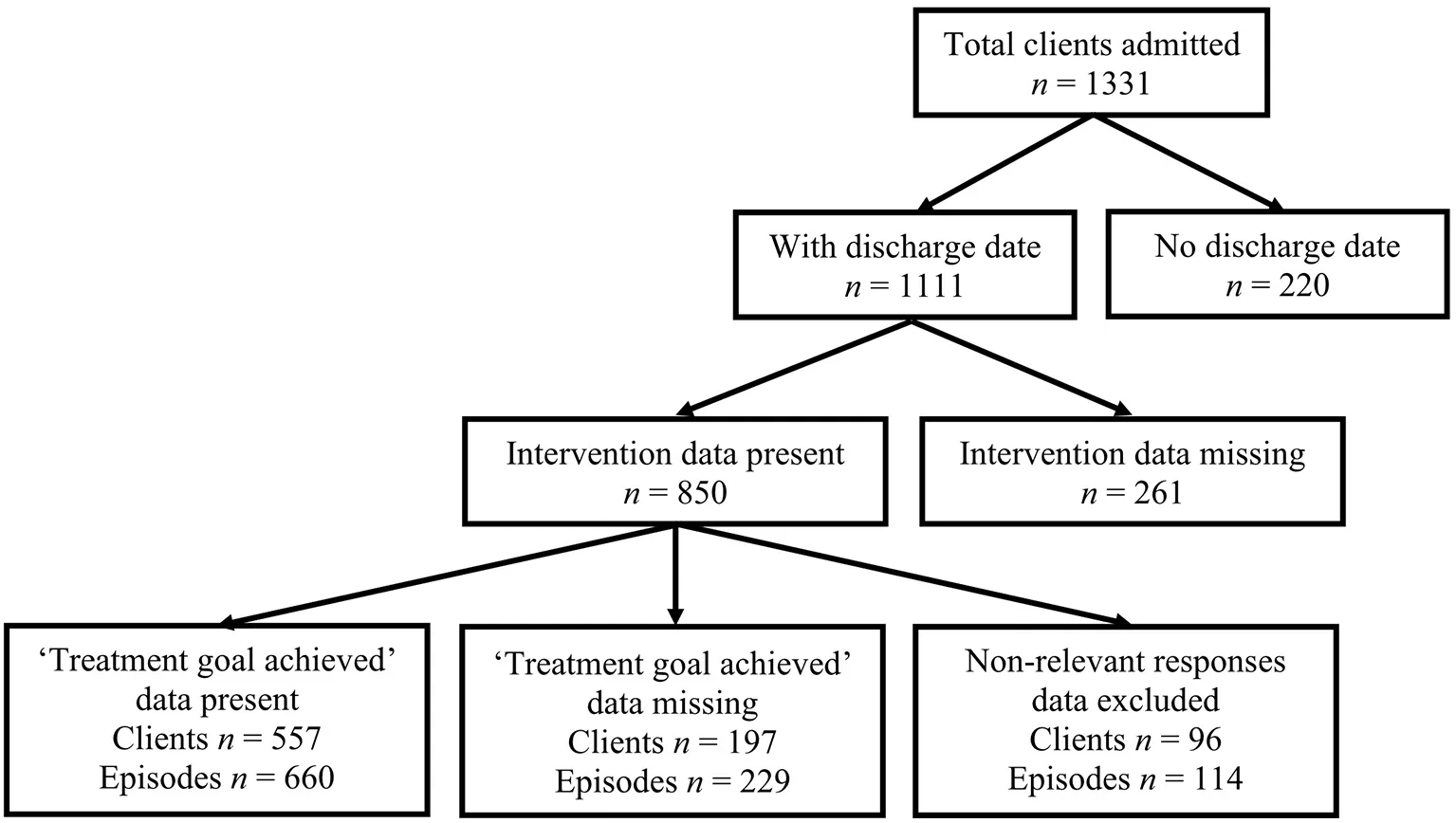
Flowchart of sample derivation for the ‘treatment goal achievement’ analysis.

**Tables 2** and **3** present the mixed-effects logistic regression analyses examining associations between EP and SP involvement and treatment completion and treatment goal achievement, respectively. Results are presented for minimally adjusted models (Multivariable 1 and Multivariable 1 imputed), which included age, sex, country of birth and IRSD decile scores, and fully adjusted models (Multivariable 2 and Multivariable 2 imputed) which additionally incorporated demographic, clinical and service-related covariates as described in the Methods and table notes.

**Table 2:** Association between exercise physiology, speech pathology and treatment completion.

Intervention	Model	*n*	OR	95% Confidence interval for OR	
				Lower	Upper
Exercise physiology	Bivariate	723	4.46**	2.21	9.02
	Multivariable 1	699	5.65**	2.41	13.23
	Multivariable 1 imputed	732	4.52**	2.27	9.04
	Multivariable 2	493	5.68**	2.10	15.37
	Multivariable 2 imputed	732	4.36**	2.22	8.55
Speech pathology	Bivariate	723	2.29*	1.14	4.62
	Multivariable 1	699	2.50*	1.18	5.33
	Multivariable 1 imputed	732	2.32*	1.17	4.60
	Multivariable 2	493	2.44*	0.93	6.36
	Multivariable 2 imputed	732	2.11*	1.09	4.07

OR, odds ratio. Multivariable 1 adjusted for age, sex, country of birth, and IRSD decile. Multivariable 2 additionally adjusted for marital status, language, Aboriginal status, employment, living arrangement, health conditions, and crow distance. Imputed models used multiple imputation by chained equations (MICE). *p<0.05; **p<0.01.

**Table 3:** Association between exercise physiology, speech pathology and treatment goal achievement.

Intervention	Model	*n*	OR	95% Confidence interval for OR	
				Lower	Upper
Exercise physiology	Bivariate	660	6.67**	2.46	18.07
	Multivariable 1	633	6.67**	2.42	18.42
	Multivariable 1 imputed	889	7.43**	2.87	19.19
	Multivariable 2	438	7.26**	2.32	22.66
	Multivariable 2 imputed	889	7.02**	2.75	17.95
Speech pathology	Bivariate	660	4.01**	1.66	9.70
	Multivariable 1	633	4.05**	1.66	9.85
	Multivariable 1 imputed	732	5.21**	1.99	13.59
	Multivariable 2	456	3.75**	1.38	10.17
	Multivariable 2 imputed	732	4.88**	1.93	12.37

OR, odds ratio. Multivariable 1 adjusted for age, sex, country of birth, and IRSD decile. Multivariable 2 additionally adjusted for marital status, language, Aboriginal status, employment, living arrangement, health conditions, and crow distance. Imputed models used multiple imputation by chained equations (MICE). **p<0.01.

Individuals who received EP and/or SP services were more likely than those who did not receive these services to successfully complete treatment and achieve or partially achieve their treatment goals. In minimally adjusted models, both EP and SP involvement were associated with higher odds of treatment completion and goal achievement (treatment completion: EP OR 5.65 [95% CI 2.41–13.23], SP OR 2.50 [95% CI 1.18–5.33]; goal achievement: EP OR 6.67 [95% CI 2.42–18.42], SP OR 4.05 [95% CI 1.66–9.85]). Similar associations were observed in fully adjusted models (treatment completion: EP OR 5.68 [95% CI 2.10–15.37], SP OR 2.44 [95% CI 0.93–6.36]; goal achievement: EP OR 7.26 [95% CI 2.32–22.66], SP OR 3.75 [95% CI 1.38–10.17]).

In a sensitivity analysis, the MICE method was employed to impute missing values in outcome variables and other covariates, including IRSD decile, crow distance, employment status, and health conditions. This process generated 10 imputed datasets (seed of 54321, 100 burn-ins). All variables present in the fully adjusted model were considered for imputation. Details pertaining to the imputation models are available in **Supplementary Tables S16**–**S19** in the online supplement to this article.

#### 3.2.2 Association between exercise physiology, speech pathology and dose (Aim 2)

For Aim 2, we examined dose effects for EP and SP treatment outcomes. For both interventions, higher contact time was associated with a higher likelihood of treatment completion (EP OR 1.89 [95% CI 1.32–2.69], SP OR 1.42 [95% CI 1.02–1.97]) and treatment goal achievement (EP OR 1.89 [95% CI 1.24–2.86], SP OR 1.56 [95% CI 1.04–2.33]). Thus, for EP, a one-unit increase in log(EP time) was associated with an 89% increase in the odds of both treatment completion and treatment goal achievement. In practical terms, doubling EP time increased the odds of these outcomes by approximately 55%. For SP, the corresponding increases were smaller but still positive: a one-unit increase in log(SP time) was associated with a 42% increase in the odds of treatment completion and a 56% increase in the odds of achieving treatment goals; doubling SP time increased these odds by approximately 30–40%. EP contact number was also positively associated with treatment completion (OR 1.65 [95% CI: 1.01–2.69]) (see **Table 4**).

**Table 4:** Association between treatment completion, treatment goal achievement, and dose of exercise physiology and speech pathology.

Dose type	Treatment completion					Treatment goal achievement				
	Treatment completed		Did not engage		OR (95% CI)	Achieved		Not achieved		OR (95% CI)
	*n*	Mean (SD)	*n*	Mean (SD)		*n*	Mean (SD)	*n*	Mean (SD)	
Exercise physiology										
Contact time (min)	72	198 (218.1)	34	84.1(150.5)	1.89 (1.32–2.69)***	92	165.3 (201)	13	45.8 (60.3)	1.89 (1.24–2.86)**
Number of contacts	72	32 (24.6)	34	21.5 (17)	1.65 (1.01–2.69)*	92	28.7 (22.8)	13	18.9 (11.4)	1.43 (0.87–2.37)
Speech pathology										
Contact time (min)	42	319.5 (477.2)	28	199.8 (634.5)	1.42 (1.02–1.97)*	57	299.1 (590.1)	9	57.8 (55.9)	1.56 (1.04–2.33)*
Number of contacts	42	33.4 (27.1)	28	21.9 (16.4)	1.67 (0.96–2.91)	57	32.1 (24.6)	9	20.2 (11.5)	1.76 (0.83–3.71)

OR, odds ratio; CI, confidence interval; SD, standard deviation. Dose variables (contact minutes and number of contacts) were log-transformed prior to analysis. ORs represent the change in odds per one-unit increase in the log-transformed dose. Both models include only those with total time > 0. *p<0.05; **p<0.01; ***p<0.001.

## 4 Discussion

Using routinely collected service data, this study examined whether involvement in exercise physiology (EP) and speech pathology (SP) services was associated with behavioural indicators of treatment engagement - treatment completion and treatment goal achievement - among adults completing community-based orders (CBOs) through the Forensic Mental Health in Community Health (FMHiCH) program.

Overall, clients who received EP and/or SP were more likely to complete treatment and to achieve their treatment goals compared to those who did not receive these interventions. In addition, greater contact time within each discipline was associated with higher likelihood of both outcomes, suggesting a dose-response pattern. These findings are consistent with the program’s underlying theory of change, in which EP and SP were intended to enhance engagement through structured, individually tailored, and non-stigmatising forms of therapeutic contact.

Differences between EP and SP effects warrant consideration. Although effect sizes for EP were somewhat larger than those for SP, these differences should be interpreted cautiously given differences in sample size. The finding that the number of EP contacts predicted treatment completion may indicate that repeated physical engagement confers motivational or behavioural advantages, although alternative explanations related to pre-existing engagement levels cannot be excluded. Dose effects for SP may have been more difficult to detect due to the smaller sample size. Overall, the convergence of the main effects and dose–response findings signals the value of specialised, structured interventions such as EP and SP and underscores the importance of ensuring clients receive sufficient treatment exposure to maximise therapeutic benefit. Importantly, both interventions were associated with improved engagement outcomes, consistent with their role as accessible and appealing forms of care designed to encourage participation.

These findings suggest that EP and SP may function as accessible entry points to engagement within a population where traditional mental health approaches may be less acceptable. Exercise-based interventions may support participation by providing an activity-oriented and non-stigmatising context for engagement, while also facilitating therapeutic interaction in a less confrontational setting. In parallel, communication difficulties - prevalent in justice-involved populations - may constrain engagement by limiting individuals’ ability to understand and participate in treatment processes (29–34, 42). Addressing these through SP may therefore enable more effective involvement in care, consistent with evidence that improved communication supports access to and participation in rehabilitation and treatment programmes (32, 33). Together, these findings are consistent with the proposition that interventions which increase both the acceptability of services and individuals’ capacity to participate may enhance engagement in forensic mental health settings.

The general descriptive analyses provide important context for interpreting the results related to EP and SP. Treatment engagement was associated with a range of demographic, social, and clinical characteristics, with employment and stable housing consistently linked to better outcomes, and homelessness and psychotic disorders associated with poorer outcomes. These findings suggest that structural stability plays a central role in enabling sustained engagement in community-based forensic mental health treatment. Individuals with secure housing and employment may have greater capacity to attend appointments, maintain routines, and engage with therapeutic processes, whereas those experiencing instability may face competing priorities that disrupt continuity of care.

Geographic findings add an additional layer of complexity. Contrary to expectations, clients with longer travel time to the service were more likely to complete treatment, with distance measures showing a similar pattern. One plausible explanation is that individuals willing to travel longer represent a more motivated, better supported externally or selectively engaged subgroup, able to prioritise treatment despite increased logistical demands. While motivation was not directly measured in this study, it remains a plausible mechanism warranting further exploration. Alternatively, clients travelling from more distant suburbs - many of which have higher socio-economic status than the service’s location in the second most disadvantaged municipality in Victoria (43) - may have had greater resources that supported treatment engagement and completion (44). Although our analysis did not support this explanation (IRSD was not related to treatment completion), limitations of the project’s scope and geographic classification methods mean that this may also be a consideration worth exploring further.

Age was associated with treatment completion, with better outcomes observed among clients aged over 41 years. This finding is consistent with developmental criminology research showing that offending behaviour typically declines with age (45, 46). Older individuals may be more motivated to disengage from offending behaviour and comply with treatment requirements, particularly if they view their involvement in the criminal justice system as approaching an endpoint. This interpretation is also consistent with clinical observations from FMHiCH staff and suggests that motivation to engage may be shaped by broader life-course processes.

Taken together, the results suggest that integrating EP and SP into community-based forensic mental health care may support treatment engagement, while also underscoring the influence of social determinants, such as secure housing and employment, and client characteristics, such as age, on engagement outcomes.

### 4.1 Strengths and limitations

The inclusion of EP and/or SP as Allied Health disciplines in community-based treatment for adults with forensic histories and moderate mental health conditions represents an innovative area in criminal, legal and mental health practice but there is a scarcity of relevant research. A key strength of this study is its use of routinely collected clinical data from a community health service, providing insights into treatment outcomes under typical service conditions.

Our study employed sophisticated statistical analytic approaches to maximise the use of this real-world service data. Comparatively, the odds ratio derived from the complete case analysis was marginally higher but with wider confidence interval than that obtained from the imputed data models. The disparity in odds ratios between the two analytical approaches may be attributed to variations in the set of observations utilised in each method. Notably, the results remained significant across all models including the sensitivity analysis indicating they are robust. The findings that EP and SP were associated with better treatment outcomes are significant contributions to this important yet under-researched area. Additionally, the inclusion of extensive socio-demographic variables enhanced the analysis by considering broader contextual factors that may influence mental health and criminal outcomes.

Nevertheless, the study has several limitations. First, the generalisability of findings is confined to the specific context of this Victorian FMHiCH service, limiting the applicability of results to other services, regions, or settings. Second, the non-randomised, observational study design restricts the ability to draw causal inferences and limits control over confounding variables. Participation in EP and SP was not uniform across the client population, with referral and uptake varying according to certain client characteristics. This suggests that access to these interventions was influenced by factors such as clinician judgement, client readiness, and capacity to engage, rather than occurring at random. Consequently, individuals receiving EP or SP may have differed systematically from those who did not receive these services, introducing the possibility of selection bias. The absence of a dedicated control group further complicates the attribution of observed outcomes solely to EP and SP services.

Third, interpretation of the dose-response findings also requires caution. Clients who accumulated more EP or SP contact hours may have been more motivated, more socially stable, or more engaged with the wider forensic program. Although the consistency of dose effects across both interventions strengthens confidence that greater therapeutic exposure is beneficial, the possibility that treatment dose served as a proxy for overall engagement cannot be excluded. Nevertheless, the convergence of the main intervention analyses and the dose-response findings suggests that EP and SP may confer benefits beyond general service contact, despite the potential influence of individual engagement tendencies.

Fourth, routinely collected health service data can often vary in its consistency and completeness. The EP and SP sample sizes were relatively small for adjusted models so that estimates, particularly for SP, should be interpreted cautiously given wide confidence intervals. As well, the two outcome variables were affected by evolving reporting protocols associated with the iterative development of the FMHiCH service. For example, prior to a review of reporting processes in September 2020, treatment goal achieved was not routinely recorded in the database, and discharge reasons included a mixture of user-generated entries from the broader health service and the 13 FMHiCH pre-determined options. Additionally, the outcome indicators reflect observable behavioural manifestations of engagement but do not capture its attitudinal or relational dimensions and some ambiguity existed in their operationalised definitions.

A final limitation is that SP services were delivered by a single clinician and EP services were delivered predominantly by a single clinician, with occasional involvement from other EPs and EP students. Consequently, the observed outcomes may partly reflect clinician-specific factors, such as therapeutic style, experience, or rapport-building, rather than the intervention models alone. This may limit the generalisability of the findings to services with different staffing structures and restrict the ability to disentangle intervention effects from clinician effects.

### 4.2 Future research

While the study’s findings provide a useful starting point for evaluating the impact of EP and SP in forensic mental health services, they emphasise the need for future research to address these limitations and offer a more nuanced understanding of the program’s effectiveness. For instance, with four other FMHiCH services in Victoria currently without EP and SP, the Victorian FMHiCH service is ideally set up to evaluate the impact of EP and SP more rigorously using a stepped-wedge cluster randomised controlled trial (cRCT). A stepped-wedge cRCT is a study design in which interventions are sequentially rolled out to different cluster groups over multiple time periods, allowing for within-group and between-group comparisons while ensuring that all groups eventually receive the intervention (47). While one or more clusters is randomly ordered to receive the intervention, the remaining cluster(s) serve as the control, until they subsequently receive the intervention. It is suggested as a pragmatic and efficient approach for service delivery evaluation in settings where there may be logical or practical constraints to data collection (48). A successful example of a stepped-wedge cRCTs is the PULSAR Secondary care study, also funded by the Victorian Government (49). In the PULSAR Secondary Care study, eighteen public and community mental health services were randomly allocated to one of two steps (9 months apart) to engage in a recovery-oriented practice staff training intervention, with data collected at three time points: baseline, and at the end of Steps 1 (after one group had received the intervention) and 2 (once both groups had received the intervention). Results showed a significant positive effect on the personal recovery of consumers (50). Future research building on these findings has the potential to significantly advance evidence-based improvements in mental health services. Such contributions could inform policy development across mental health and justice sectors, not only within Victoria but also at national and international levels - ultimately promoting individual well-being and fostering a healthier, more equitable society.

## Data Availability

The data underlying this study cannot be shared publicly due to legal, ethical, and privacy restrictions. The dataset comprises retrospective health service records co-held by the Forensic Mental Health in Community Health (FMHiCH) program, the Department of Health, and the Department of Justice and Community Safety. In accordance with the National Health and Medical Research Council (NHMRC) guidelines (2018), a waiver of individual consent was granted because it was not practicable to obtain consent for all cases. The Monash Health Research Office reviewed and approved the project via an alternate pathway, confirming exemption from HREC review. Requests to access the datasets can be directed to the corresponding author; however access is subject to the aforementioned legal, ethical and privacy restrictions.
