# Supplementary material for: Exercise physiology and speech pathology support treatment engagement in forensic mental health in community health: evidence from a retrospective cohort study
Source: Front Psychiatry. 2026 Oct 19;17:1964444. doi: 10.3389/fpsyt.2026.1964444 (PMC13724423; doi:10.3389/fpsyt.2026.1964444)
Supplement: Supplementary file 1 [file DataSheet1.pdf]

Online Supplementary Material for:

Exercise Physiology and Speech Pathology Support Treatment Engagement in Forensic Mental Health in Community Health: Evidence from a Retrospective Cohort Study

Table of Contents

S1 Supplementary methods 2
S1.1 Detailed literature search strategies 2
S1.2 Search strategy tables 3
Table S1 Original literature review search terms 3
Table S2 Updated OVID MEDLINE (ALL) search strategy 4
Table S3 Updated CINAHL search strategy 7
S2 Supplementary results tables 10
S2.1 Confidentiality and data presentation 10
Table S4 Categorization of ‘reason for discharge’ 11
Table S5 Categorization of ‘treatment goal achieved’ 11
Table S6 Client characteristics by treatment completion status (client-level data) 12
Table S7 Client characteristics by treatment completion status (episode-level data) 16
Table S8 Client characteristics by treatment goal achievement status (client-level data) 20
Table S9 Client characteristics by treatment goal achievement status (episode-level data) 24
Table S10 Treatment completion (discharge reason) status by group 28
Table S11 Treatment goal achievement status by group 28
Table S12 Client characteristics by exercise physiology participation status (client-level data) 29
Table S13 Client characteristics by exercise physiology participation status (episode-level data) 33
Table S14 Client characteristics by speech pathology participation status (client-level data) 37
Table S15 Client characteristics by speech pathology participation status (episode-level data) 41
Table S16 Conditional imputation models for the analysis of discharge reasons 45
Table S17 Results from imputation (outcome: completed treatment) 46
Table S18 Conditional imputation models for the analysis of treatment goals 47
Table S19 Results from imputation (outcome: treatment goals) 48

S1 Supplementary methods

S1.1 Detailed literature search strategies

S1.1.1 Original literature search strategy

Between July and August 2022, the OVID MEDLINE and CINAHL databases were searched using terms set out in Table 1 below, truncated and combined variously with BOOLEAN operators “AND” and “OR” and searched both as keywords and mapped to subject headings in the search tool where applicable. Date parameters for the search were not employed to uncover as much relevant material as possible. Minor exploratory searching of the ProQuest Criminology and Criminal Justice databases were also conducted at this point to determine whether to expand the search into these collections. Initial results returned did not appear to yield any new relevant titles and as such these databases were not explored further.

S1.1.2 Updated literature search strategy

A comprehensive literature search was conducted to identify studies published from 2022 involving exercise-based and speech-based interventions in community service forensic populations. The search strategy was developed in collaboration with a specialist librarian from Monash University with expertise in high-quality literature searches. It was built using a combination of subject headings (MeSH) and keywords, organized into four conceptual groups: (1) Criminal Justice /Forensic/ Legal, (2) Community/Mental Health Services, (3) Exercise, and (4) Speech/Language/Communication. The primary focus was on exercise-based and speech-language interventions for individuals with mental health conditions, accessing services in community settings. Mental health disorders were not included as a separate conceptual group due to their broad scope and variability; instead, they were considered implicitly through the inclusion of community mental health services. The search conducted on 23/9/25 using the OVID MEDLINE ALL (“Ovid MEDLINE(R) ALL 1946 to September 19, 2025”) and CINAHL databases. Boolean operators were used to combine terms within and across groups. The final searches combined all concepts by “AND” and limited to articles published from 2022 to current (19/9/2025 for MEDLINE and 23/9/2025 for CIHAHL).

Inclusion criteria: population: forensic clients with mental health conditions accessing community health services; intervention: speech pathology-related interventions or exercise-related interventions

Exclusion criteria: prison populations

S1.2 Search strategy tables

Table S1 Original literature review search terms

• communication	• exercise	• mental	• scien*
• community	• forensic	• offen*	• speech
• crime	• health	• path*	• speech-language
• criminal	• impair*	• pathology	• therap*
• dis*	• justice	• physiolog*	• therapy
• disab*	• language	• psychiatry	• voice
• disabilities	• law	• psychology	
• disorders	• learning	• public	

Note: search terms were truncated and combined variously with BOOLEAN operators "AND" and "OR" and searched both as keywords and mapped to subject headings in the search tool where applicable.

Table S2 Updated OVID MEDLINE (ALL) search strategy

Conceptual Group	Mapped Subject Headings (MeSH)	Keywords
Criminal Justice / Forensic / Legal	Criminals, Recidivism, Criminal Law, Forensic Psychiatry, Forensic Psychology, Criminal Psychology, Criminal Behavior, Crime<br><br>S1 Criminals/ or Recidivism/ or Criminal Law/ or Forensic Psychiatry/ or Forensic Psychology/ or Criminal Psychology/ or Criminal Behavior/ or Crime/<br><br>S3: S1 OR S2	criminal*, crime, justice, forensic psych*, law, offen*, recidiv*, parole, legal, repeat offend*, public offend*<br><br>S2 (criminal* or crime or justice or forensic psych* or law or offend* or recidiv* or parole or legal or repeat offend* or public offend*).mp.
Community / Mental Health Services	Community Psychiatry, Community Health Centers, Community Health Services, Community Mental Health Services, Community Mental Health Centers<br><br>S4 Community Psychiatry/ or Community Health Centers/ or Community Health Services/ or Community Mental Health Services/ or Community Mental Health Centers/<br><br>S6: S4 OR S5<br>S7: S3 AND S6	community health*, community mental health*, community psych*, community program*<br><br>S5 (Community health* or Community mental health* or Community psych* or Community program*).mp.
Speech/ Language/Communication	Communication Disorders, Language Disorders, Learning Disabilities, Speech Disorders, Speech Therapy, Speech-Language Pathology, Voice Disorders,<br>Language Development Disorders, Language Therapy, Rehabilitation of Speech and Language Disorders	Speech pathol*<br>Speech therap*<br>Speech-language pathol*<br>Communication disorder*<br>Language disorder*<br>Speech disorder*<br>Voice disorder*<br>Language development disorder*<br>Language impairment*<br>Language problem*<br>Language difficult*<br>Language disab*<br>Communication difficult*

		Communication impairment*<br>Communication problem*<br>learning disab*<br>Learning disorder*<br>Language therap*<br>Speech impairment*<br>Speech problem*<br>Speech difficult*
	S8 Communication Disorders/ or Language Disorders/ or Learning Disabilities/ or Speech Disorders/ or Speech Therapy/ or Speech-Language Pathology/ or Voice Disorders/ or Language Development Disorders/ or Language Therapy/<br><br>S9 Rehabilitation of Speech and Language Disorders mapped to subject heading	S10 (Speech pathol* or Speech therap* or Speech-language pathol* or Speech disorder* or Speech impairment* or Speech problem* or Speech difficult*).mp.<br><br>S11 (Language disorder* or Language development disorder* or Language impairment* or Language problem* or Language difficult* or Language disab* or learning disab* or learning disorder* or Language therap*).mp.<br><br>S12 (Communication disorder* or Voice disorder* or Communication difficult* or Communication impairment* or Communication problem*).mp.
	S13: S8 or S9 or S10 or S11 or S12	
Exercise	Exercise Therapy, Physical Fitness, Physical Therapists	Exercise therap*<br>Exercise physiolog*<br>Exercise scien*<br>Physical therap*<br>Exercise*<br>Physical activit*<br>Physical training<br>Exercise training<br>therapeutic exercise*<br>physical fitness

	S14 Exercise Therapy/ or Physical Fitness/ or Physical Therapists/	S15(Exercise therap* or Exercise physiolog* or Exercise scien* or Physical therap* or exercise or Physical activit* or Physical training or Exercise training or therapeutic exercise* or physical fitness).mp.
	S16: S14 OR S15<br>S17: S13 OR S16<br>S18: S7 AND S17<br>S19: limit 18 to yr="2022 -Current"	

Table S3 Updated CINAHL search strategy

Conceptual Group	Mapped Subject Headings (MeSH)	Keywords
Criminal Justice / Forensic / Legal	Crime, Recidivism, Criminal Justice, Criminal Psychology, Forensic Psychology, Repeat Offenders, Public Offenders, Forensic Psychiatry<br>S1 (MH "Crime" OR MH "Recidivism" OR MH "Criminal Justice" OR MH "Criminal Psychology" OR MH "Forensic Psychology" OR MH "Repeat Offenders" OR MH "Public Offenders" OR MH "Forensic Psychiatry")<br>S3: S1 OR S2	criminal*, crime, justice, forensic psych*, law, offen*, recidiv*, parole, legal, repeat offend*, public offend*<br>S2 "criminal*" OR "crime" OR "justice" OR "forensic psych*" OR "law" OR "offen*" OR "recidiv*" OR "parole" OR "legal" OR "repeat offend*" OR "public offend*"
Community / Mental Health Services	Community Health Centers, Community Health Services, Community Programs, Community Mental Health Services<br>S4 MH "Community Health Centers" OR MH "Community Health Services" OR MH "Community Programs" OR MH "Community Mental Health Services"<br>S7: S4 OR S6	community health*, community mental health*, community psych*, community program*<br>S5 "Community health*" OR "Community mental health*" OR "Community psych*" OR "Community program*"
Speech/ Language/Communication	Communicative Disorders, Language Disorders, Speech Disorders, Voice Disorders, Learning Disorders, Speech Therapy, Rehabilitation, Speech and Language, Language Therapy, Speech Disorders, Speech-Language Pathology	Speech pathol*<br>Speech therap*<br>Speech-language pathol*<br>Communication disorder*<br>Language disorder*<br>Speech disorder*<br>Voice disorder*<br>Language development disorder*<br>Language impairment*<br>Language problem*<br>Language difficult*<br>Language disab*<br>Communication difficult*<br>Communication impairment*<br>Communication problem*<br>learning disab*

		Learning disorder*<br>Language therap*<br>Speech impairment*<br>Speech problem*<br>Speech difficult*
	S7 MH "Communicative Disorders" OR MH "Language Disorders" OR MH "Speech Disorders" OR MH "Voice Disorders" OR MH "Learning Disorders" OR MH "Speech Therapy" OR MH "Rehabilitation, Speech and Language" OR MH "Language Therapy" OR MH "Speech Disorders" OR MH "Speech-Language Pathology"	S8 "speech pathol*" OR "speech therap*" OR "Speech-language pathol*" OR "speech disorder*" OR "speech impairment*" OR "speech problem*" OR "speech difficult*"<br><br>S9 "language disorder*" OR "language development disorder*" OR "language impairment*" OR "language problem*" OR "language difficult*" OR "language disab*" OR "language therap*" OR "learning disab*" OR "learning disorder*"<br><br>S10 "communication difficult*" OR "communication impairment*" OR "communication problem*" OR "voice disorder*" OR "communication disorder*"
	S11: S7 OR S8 OR S9 OR S10	
Exercise	Therapeutic Exercise, Exercise, Physical Therapy, Exercise Physiologists, Exercise Physiology, Physical Fitness, Physical Therapists, Physical Activity	exercise therap*, exercise physiolog*, exercise scien*, physical therap*, exercise*, physical activit*, physical training, exercise training, therapeutic exercise*, physical fitness
	S12 MH "Therapeutic Exercise" OR MH "Exercise" OR MH	S13 "exercise therap*" OR "exercise physiolog*" OR

	"Physical Therapy" OR MH "Exercise Physiologists" OR MH "Exercise Physiology" OR MH "Physical Fitness" OR MH "Physical Therapists" OR MH "Physical Activity"	"exercise scien*" OR "physical therap*" OR "exercise*" OR "physical activit*" OR "physical training" OR "exercise training" OR "therapeutic exercise*" OR "physical fitness"
	S14: S12 OR S13	
	S15: S11 OR S14	
	S16: S3 AND S6	
	S17: S15 AND S16	
	S18: S17 With filter	
	Publication Date (start date)	2022-01-01
	Publication Date (end date)	2025-09-23

S2 Supplementary results tables

S2.1 Confidentiality and data presentation

To protect participant confidentiality, small cell sizes were managed using targeted suppression. Categories with very small counts (e.g. <5) were suppressed (denoted as "–") where there was potential for re-identification, particularly for sensitive variables (e.g. refugee status, interpreter use, and detailed diagnostic categories). Totals were also suppressed where necessary to prevent back-calculation of small cells. Non-sensitive administrative categories (e.g. missing data) were retained where disclosure risk was negligible.

Table S4 Categorization of 'reason for discharge'

'Discharge Reason' Label	Incorporated 'Discharge Reason' Outcomes
Completed treatment	Client no longer needs assistance – improved status
	Client no longer needs assistance from agency - improved status
	Client no longer requires assistance
	Completed Treatment
	Completed treatment/therapy
Did not engage	Client terminated service
	Did not complete treatment/therapy
	Disengaged
	Refused
	Unable to make initial contact
Became unavailable	Admission to hospital/rehabilitation
	Client died
	Client has moved out of area
	Client moved to other community-based service
	Client moved to other institutional setting
	Client now requires higher level aged care
	Deceased
	Incarcerated/on remand
	Moved out of area
	Step-up to Area Mental Health Service
	Supervision transferred to another CCS office
No treatment required	No mental health treatment required
	Service unable to provide assistance

Note. Categorization was conducted in consultation with FMHiCH staff. Categories were grouped to distinguish treatment completion from non-engagement, with discharge reasons unrelated to treatment engagement (e.g., relocation, hospital admission) excluded from regression analyses.

Table S5 Categorization of 'treatment goal achieved'

New 'Treatment Goal' Label	Incorporated 'Treatment Goal' Outcomes
Treatment goal achieved/partially achieved	Treatment goal achieved
	Treatment goal partially achieved
Treatment goal not achieved	Treatment goal not achieved
	Client refused assessment/did not consent/disengaged
Other	Unable to make initial contact
	Other/blank

Note. Categorization was conducted in consultation with FMHiCH staff. Treatment goal achieved" and "treatment goal partially achieved" were combined because the distinction between partial and full achievement lacked standardised criteria and partial achievement was generally considered to represent meaningful progress towards individualised treatment goals.

Table S6 Client characteristics by treatment completion status (client-level data)

Variable	*N*	Completed treatment *n* (%)	Did not engage *n* (%)	χ^2 (*V*)	*p*-value
Country of birth					
Australia	585 (81.5)	270 (77.8)	315 (84.9)		
Asia	56 (7.8)	38 (11.0)	18 (4.9)		
Oceania	26 (3.6)	15 (4.3)	11 (3.0)		
Europe	20 (2.8)	11 (3.2)	9 (2.4)		
Africa and others	31 (4.3)	13 (3.7)	18 (4.9)		
**Total**	**718**	**347 (100)**	**371 (100)**	11.44 (0.13)	0.02*
Age category					
18-25	146 (20.0)	59 (16.9)	87 (22.9)		
26-40	396 (54.2)	180 (51.4)	216 (56.8)		
41 and above	188 (25.8)	111 (31.7)	77 (20.3)		
**Total**	**730**	**350 (100)**	**380 (100)**	13.58 (0.14)	0.001**
Sex					
Male	585 (80.1)	281 (80.3)	304 (80.0)		
Female	145 (19.9)	69 (19.7)	76 (20.0)		
**Total**	**730**	**350 (100)**	**380 (100)**	0.009 (0.004)	0.92
Marital status					
Single	487 (66.7)	207 (59.1)	280 (73.7)		
De facto	88 (12.1)	54 (15.4)	34 (8.9)		
Divorced	22 (3.0)	13 (3.7)	9 (2.4)		
Married	40 (5.5)	27 (7.7)	13 (3.4)		
Not known	70 (9.6)	36 (10.3)	34 (8.9)		
Separated	23 (3.2)	13 (3.7)	10 (2.6)		
**Total**	**730**	**350 (100)**	**380 (100)**	20.37 (0.17)	0.001**
Preferred language					
English	706 (97.0)	335 (95.7)	371 (98.1)		
Others	22 (3.0)	15 (4.3)	7 (1.9)		
**Total**	**728**	**350 (100)**	**378 (100)**	3.67 (0.07)	0.06

Variable	N	Completed treatment n (%)	Did not engage n (%)	χ^2 (V)	p-value
Aboriginal status					
Not Indigenous	673 (92.2)	330 (94.3)	343 (90.3)		
Aboriginal or TSI	44 (6.0)	13 (3.7)	31 (8.2)		
Declined to answer	13 (1.8)	7 (2.0)	6 (1.6)		
**Total**	**730**	**350 (100)**	**380 (100)**	6.47 (0.09)	0.04*
Employment status					
Unemployed	385 (70.8)	173 (65.3)	212 (76.0)		
Employed	143 (26.3)	84 (31.7)	59 (21.2)		
Other occupation	16 (2.9)	8 (3.0)	8 (2.9)		
**Total**	**544**	**265 (100)**	**279 (100)**	7.97 (0.12)	0.02*
Refugee status					
Not a refugee or asylum seeker	684 (99.6)	326 (99.7)	358 (99.4)		
Refugee or asylum seeker	-	-	-		
**Total**	-	-	-	0.25 (0.02)	0.62
Carer status					
Does not have carer	36 (76.6)	19 (79.2)	17 (73.9)		
Has a carer	-	-	-		
**Total**	-	-	-	0.18 (0.06)	0.67
Living arrangement					
Lives alone	59 (8.1)	31 (8.9)	28 (7.4)		
Lives with family	294 (40.3)	150 (42.9)	144 (37.9)		
Lives with others	155 (21.2)	73 (20.9)	82 (21.6)		
Not stated	222 (30.4)	96 (27.4)	126 (33.2)		
**Total**	**730**	**350 (100)**	**380 (100)**	3.63 (0.07)	0.31
Accommodation type					
Secure housing	293 (75.7)	157 (80.9)	136 (70.5)		
Homeless	28 (7.2)	7 (3.6)	21 (10.9)		
Emergency/transitional housing	11 (2.8)	5 (2.6)	6 (3.1)		
Other	55 (14.2)	25 (12.9)	30 (15.5)		
**Total**	**387**	**194 (100)**	**193 (100)**	9.05 (0.15)	0.03*

**Variable**	***N***	**Completed treatment *n* (%)**	**Did not engage *n* (%)**	χ^2 (*V*)	***p*-value**
Need for interpreter services					
No interpreter needed	715 (98.9)	340 (98.3)	375 (99.5)		
Interpreter needed	-	-	-		
**Total**	-	-	-	2.39 (0.06)	0.12
Health conditions					
Mental illness	239 (41.9)	115 (42.4)	124 (41.3)		
Alcohol & other drugs	298 (52.2)	136 (50.2)	162 (54.0)		
Physical illness or injury	29 (5.1)	15 (5.5)	14 (4.7)		
Others	5 (0.9)	5 (1.8)	0 (0.0)		
**Total**	**571**	**271 (100)**	**300 (100)**	6.19 (0.10)	0.10
Mental health conditions					
F40 - F49	152 (41.8)	82 (45.6)	70 (38.0)		
F00 – F09	-	-	-		
F10 - F19	13 (3.6)	7 (3.9)	6 (3.3)		
F20 - F29	-	-	-		
F30 - F39	146 (40.1)	72 (40.0)	74 (40.2)		
F50 - F59	-	-	-		
F60 - F69	11 (3.0)	5 (2.8)	6 (3.3)		
F70 - F79	-	-	-		
F80 - F89	-	-	-		
F90 - F98	-	-	-		
F99	-	-	-		
**Total**	**364**	**180 (100)**	**184 (100)**	14.27 (0.20)	0.16
IRSD*					
< 50th percentile	331 (46.0)	151 (43.4)	180 (48.4)		
≥ 50th percentile	389 (54.0)	197 (56.6)	192 (51.6)		
**Total**	**720**	**348 (100)**	**372 (100)**	1.81 (0.18)	0.18

Variable	*N*	Completed treatment *n* (%)	Did not engage *n* (%)	χ^2 (*V*)	*p*-value
Crow distance category					
< 4 kms	224 (31.2)	94 (27.1)	130 (34.9)		
4-9 kms	250 (34.8)	130 (37.5)	120 (32.3)		
> 9 kms	245 (34.1)	123 (35.4)	122 (32.8)		
**Total**	**719**	**347 (100)**	**372 (100)**	5.33 (0.09)	0.07
Land distance category					
< 7 kms	213 (29.6)	87 (25.1)	126 (33.9)		
7-11 kms	261 (36.3)	137 (39.5)	124 (33.3)		
> 11 kms	245 (34.1)	123 (35.4)	122 (32.8)		
**Total**	**719**	**347 (100)**	**372 (100)**	6.93 (0.10)	0.03*
Travel time (minutes)					
< 9 mins	193 (26.8)	75 (21.6)	118 (31.7)		
9-15 mins	281 (39.1)	149 (42.9)	132 (35.5)		
> 15 mins	245 (34.1)	123 (35.4)	122 (32.8)		
**Total**	**719**	**347 (100)**	**372 (100)**	9.76 (0.12)	0.008**

Notes. Univariate chi-square analyses using unique client-level data. Totals vary due to missing data; values are reported based on the number of non-missing observations (*n*) for each variable. Percentages for *N* are column percentages of the total sample. Percentages for outcome columns are calculated within outcome groups. Percentages may not equal 100 based on rounding.
*p<0.05; **p<0.01.

TSI = Torres Strait Islander. IRSD = Index of Relative Socio-economic Disadvantage. Other occupation = Home duties, Student, Other. *V* = Cramer's V effect size.

The codes listed under the 'mental health conditions' variable correspond to the groupings of mental and behavioural disorders as outlined in the International Classification of Diseases, 10th revision (ICD-10). They are as follows:

F00-F09	Organic, including symptomatic, mental disorders
F10-F19	Mental and behavioural disorders due to psychoactive substance use
F20-F29	Schizophrenia, schizotypal and delusional disorders
F30-F39	Mood [affective] disorders
F40-F49	Neurotic, stress-related and somatoform disorders
F50-F59	Behavioural syndromes associated with physiological disturbances & physical factors
F60-F69	Disorders of adult personality and behaviour
F70-F79	Mental retardation
F80-F89	Disorders of psychological development
F90-F98	Behavioural and emotional disorders with onset usually occurring in childhood and adolescence
F99	Unspecified mental disorder

Table S7 Client characteristics by treatment completion status (episode-level data)

Variable	*N*	Completed treatment *n* (%)	Did not engage *n* (%)	OR (95% CI)	*p*-value
Country of birth					
Australia (ref)	679 (81.7)	304 (78.1)	375 (84.8)		
Asia	63 (7.6)	42 (10.8)	21 (4.8)	3.24 (1.46-7.19)	0.004*
Oceania	34 (4.1)	16 (4.1)	18 (4.1)	1.21 (0.48-3.07)	0.69
Europe	23 (2.8)	13 (3.3)	10 (2.3)	1.89 (0.62-5.81)	0.26
Africa and others	32 (3.9)	14 (3.6)	18 (4.1)	0.89 (0.35-2.28)	0.81
**Total**	**831**	**389 (100)**	**442 (100)**		
Age category					
18-25 (ref)	175 (20.7)	74 (18.8)	101 (22.3)		
26-40	461 (54.5)	200 (50.8)	261 (57.7)	1.08 (0.70-1.67)	0.72
41 and above	210 (24.8)	120 (30.5)	90 (19.9)	2.06 (1.20-3.54)	0.008**
**Total**	**846**	**394 (100)**	**452 (100)**		
Sex					
Male (ref)	683 (80.7)	318 (80.7)	365 (80.8)		
Female	163 (19.3)	76 (19.3)	87 (19.2)	1.00 (0.65-1.55)	0.99
**Total**	**846**	**394 (100)**	**452 (100)**		
Marital status					
Single (ref)	574 (67.8)	240 (60.9)	334 (73.9)		
De facto	101 (11.9)	57 (14.5)	44 (9.7)	2.00 (1.16-3.46)	0.01*
Divorced	22 (2.6)	13 (3.3)	9 (2.0)	2.23 (0.79-6.33)	0.13
Married	43 (5.1)	28 (7.1)	15 (3.3)	2.99 (1.33-6.72)	0.008**
Not known	76 (9.0)	39 (9.9)	37 (8.2)	1.54 (0.86-2.75)	0.15
Separated	30 (3.5)	17 (4.3)	13 (4.3)	1.92 (0.78-4.74)	0.15
**Total**	**846**	**394 (100)**	**452 (100)**		
Preferred language					
English (ref)	820 (97.2)	378 (95.9)	442 (98.2)		
Others	24 (2.8)	16 (4.1)	8 (1.8)	3.02 (0.96-9.48)	0.06
**Total**	**844**	**394 (100)**	**450 (100)**		

Variable	*N*	Completed treatment *n* (%)	Did not engage *n* (%)	OR (95% CI)	*p*-value
Aboriginal status					
Not Indigenous (ref)	780 (92.2)	371 (94.2)	409 (90.5)		
Aboriginal or TSI	52 (6.1)	16 (4.1)	36 (8.0)	0.41 (0.19-0.91)	0.03
Declined to answer	14 (1.7)	7 (1.8)	7 (1.5)	1.18 (0.31-4.52)	0.82
**Total**	**846**	**394 (100)**	**452 (100)**		
Employment status					
Unemployed (ref)	463 (72.3)	200 (66.0)	263 (78.0)		
Employed	158 (24.7)	94 (31.0)	64 (19.0)	2.46 (1.36-4.46)	0.003**
Other occupation	19 (3.0)	9 (3.0)	10 (3.0)	1.20 (0.32-4.55)	0.79
**Total**	**640**	**303 (100)**	**337 (100)**		
Refugee status					
Not a refugee or asylum seeker (ref)	797 (99.5)	370 (99.7)	427 (99.3)		
Refugee or asylum seeker	-	-	-	0.35 (0.02-5.42)	0.45
**Total**	-	-	-		
Carer status					
Does not have carer (ref)	42 (76.4)	22 (81.5)	20 (71.4)		
Has a carer	13 (23.6)	5 (18.5)	8 (28.6)	Insufficient data	
**Total**	**55**	**27 (100)**	**28 (100)**		
Living arrangement					
Lives alone (ref)	68 (8.0)	33 (8.4)	35 (7.7)		
Lives with family	345 (40.8)	174 (44.2)	171 (37.8)	1.12 (0.57-2.20)	0.74
Lives with others	188 (22.2)	84 (21.3)	104 (23.0)	0.82 (0.40-1.68)	0.58
Not stated	245 (29.0)	103 (26.1)	142 (31.4)	0.71 (0.35-1.43)	0.33
**Total**	**846**	**394 (100)**	**452 (100)**		
Accommodation type					
Secure housing (ref)	345 (76.0)	176 (80.4)	169 (71.9)		
Homeless	32 (7.0)	8 (3.7)	24 (10.2)	0.21 (0.06-0.74)	0.015*
Emergency/transitional housing	11 (2.4)	5 (2.3)	6 (2.6)	0.70 (0.13- 3.81)	0.68
Other	66 (14.5)	30 (13.7)	36 (15.3)	0.72 (0.33-1.55)	0.40
**Total**	**454**	**219 (100)**	**235 (100)**		

**Variable**	***N***	**Completed treatment *n* (%)**	**Did not engage *n* (%)**	**OR (95% CI)**	***p*-value**
Need for interpreter services					
No interpreter needed (ref)	834 (99.0)	384 (98.5)	450 (99.6)		
Interpreter needed	-	-	-	4.90 (0.66-36.61)	0.12
**Total**	-	-	-		
Health conditions					
Mental illness (ref)	284 (42.5)	136 (43.9)	148 (41.3)		
Alcohol & other drugs	347 (51.9)	152 (49.0)	195 (54.5)	0.85 (0.58-1.23)	0.38
Physical illness or injury	32 (4.8)	17 (5.5)	15 (4.2)	1.34 (0.55-3.27)	0.52
Others	5 (0.7)	5 (1.6)	0 (0.0)	Insufficient data	
**Total**	**668**	**310 (100)**	**358 (100)**		
Mental health conditions					
F40 - F49 (ref)	179 (42.6)	97 (46.9)	82 (38.5)		
F00 – F09	-	-	-	Insufficient data	
F10 - F19	13 (3.1)	7 (3.4)	6 (2.8)	0.99 (0.32-3.05)	0.98
F20 - F29	18 (4.3)	5 (2.4)	13 (6.1)	0.33 (0.11-0.95)	0.04*
F30 - F39	166 (39.5)	81 (39.1)	85 (39.9)	0.81 (0.53-1.23)	0.32
F50 - F59	-	-	-	Insufficient data	
F60 - F69	14 (3.3)	6 (2.9)	8 (3.8)	0.63 (0.21-1.90)	0.42
F70 - F79	-	-	-	Insufficient data	
F80 - F89	-	-	-	1.69 (0.15-18.98)	0.67
F90 - F98	-	-	-	0.23 (0.06-0.85)	0.03*
F99	-	-	-	0.36 (0.09-1.45)	0.15
**Total**	**420**	**207 (100)**	**213 (100)**		
IRSD*					
< 50th percentile (ref)	391 (46.9)	169 (43.3)	222 (50.1)		
≥ 50th percentile	442 (53.1)	221 (56.7)	221 (49.9)	1.43 (0.99-2.06)	0.06
**Total**	**833**	**390 (46.8)**	**443 (53.2)**		

Variable	*N*	Completed treatment *n* (%)	Did not engage *n* (%)	OR (95% CI)	*p*-value
Crow distance category					
< 4 kms (ref)	263 (31.6)	107 (27.5)	156 (35.2)		
4-9 kms	293 (35.2)	148 (38.0)	145 (32.7)	1.71 (1.07-2.75)	0.03*
> 9 kms	276 (33.2)	134 (34.4)	142 (32.1)	1.54 (0.97-2.45)	0.07
**Total**	**832**	**389 (100)**	**443 (100)**		
Land distance category					
< 7 kms (ref)	248 (29.8)	99 (25.4)	149 (33.6)		
7-11 kms	308 (37.0)	156 (40.1)	152 (34.3)	1.79 (1.11-2.89)	0.02*
> 11 kms	276 (33.2)	134 (34.4)	142 (32.1)	1.60 (1.00-2.57)	0.05
**Total**	**832**	**389 (100)**	**443 (100)**		
Travel time (minutes)					
< 9 mins (ref)	228 (27.4)	87 (22.4)	141 (31.8)		
9-15 mins	328 (39.4)	168 (43.2)	160 (36.1)	2.03 (1.23-3.35)	0.006**
> 15 mins	276 (33.2)	134 (34.4)	142 (32.1)	1.76 (1.07-2.89)	0.03*
**Total**	**832**	**389 (100)**	**443 (100)**		

Notes. Bivariate logistic regression analyses using episode-level data. Reference category listed first and marked with (ref) in the OR calculations. Totals vary due to missing data; values are reported based on the number of non-missing observations (*n*) for each variable. Percentages for N are column percentages of the total sample. Percentages for outcome columns are calculated within outcome groups. Percentages may not equal 100 based on rounding. * p<0.05.

TSI = Torres Strait Islander. IRSD = Index of Relative Socio-economic Disadvantage. OR = Odds ratio. CI = Confidence interval. Other occupation = Home duties, Student, Other.

The codes listed under the 'mental health conditions' variable correspond to the groupings of mental and behavioural disorders as outlined in the International Classification of Diseases, 10th revision (ICD-10). They are as follows:

F00-F09	Organic, including symptomatic, mental disorders
F10-F19	Mental and behavioural disorders due to psychoactive substance use
F20-F29	Schizophrenia, schizotypal and delusional disorders
F30-F39	Mood [affective] disorders
F40-F49	Neurotic, stress-related and somatoform disorders
F50-F59	Behavioural syndromes associated with physiological disturbances & physical factors
F60-F69	Disorders of adult personality and behaviour
F70-F79	Mental retardation
F80-F89	Disorders of psychological development
F90-F98	Behavioural and emotional disorders with onset usually occurring in childhood and adolescence
F99	Unspecified mental disorder

Table S8 Client characteristics by treatment goal achievement status (client-level data)

Variable	*N*	Goal achieved *n* (%)	Goal not achieved *n* (%)	χ^2 (*V*)	*p*-value
Country of birth					
Australia	460 (82.0)	314 (81.3)	146 (83.4)		
Asia	37 (6.6)	30 (7.8)	7 (4.0)		
Oceania	25 (4.5)	18 (4.7)	7 (4.0)		
Europe	-	-	-		
Africa and others	23 (4.1)	12 (3.1)	11 (6.3)		
**Total**	-	-	-	6.03 (0.10)	0.20
Age category					
18-25	114 (20.0)	70 (17.8)	44 (24.9)		
26-40	310 (54.3)	214 (54.3)	96 (54.2)		
41 and above	147 (25.7)	110 (27.9)	37 (20.9)		
**Total**	**571**	**394 (100)**	**177 (100)**	5.41 (0.10)	0.07
Sex					
Male	464 (81.3)	320 (81.2)	144 (81.4)		
Female	107 (18.7)	74 (18.8)	33 (18.6)		
**Total**	**571**	**394 (100)**	**177 (100)**	0.002 (0.002)	0.97
Marital status					
Single	380 (66.5)	253 (64.2)	127 (71.8)		
De facto	69 (12.1)	54 (13.7)	15 (8.5)		
Divorced	-	-	-		
Married	25 (4.4)	19 (4.8)	6 (3.4)		
Not known	64 (11.2)	45 (11.4)	19 (10.7)		
Separated	18 (3.2)	11 (2.8)	7 (4.0)		
**Total**	-	-	-	5.81 (0.10)	0.33
Preferred language					
English	556 (97.4)	385 (97.7)	171 (96.6)		
Others	15 (2.6)	9 (2.3)	6 (3.4)		
**Total**	**571**	**394 (100)**	**177 (100)**	0.58 (0.03)	0.45

Variable	N	Goal achieved n (%)	Goal not achieved n (%)	χ^2 (V)	p-value
Aboriginal status					
Not Indigenous	541 (94.7)	376 (95.4)	165 (93.2)		
Aboriginal or TSI	24 (4.2)	13 (3.3)	11 (6.2)		
Declined to answer	6 (1.1)	5 (1.3)	1 (0.6)		
**Total**	**571**	**394 (100)**	**177 (100)**	3.11 (0.07)	0.21
Employment status					
Unemployed	352 (72.3)	229 (67.8)	123 (82.6)		
Employed	123 (25.3)	99 (29.3)	24 (16.1)		
Other occupation	-	-	-		
**Total**	-	-	-	11.35 (0.15)	0.003**
Refugee status					
Not a refugee or asylum seeker	540 (99.4)	375 (99.7)	165 (98.8)		
Refugee or asylum seeker	-	-	-		
**Total**	-	-	-	1.83 (0.06)	0.18
Carer status					
Does not have carer	29 (82.9)	16 (80.0)	13 (86.7)		
Has a carer	-	-	-		
**Total**	-	-	-	0.27 (0.09)	0.61
Living arrangement					
Lives alone	53 (9.3)	34 (8.6)	19 (10.7)		
Lives with family	267 (46.8)	187 (47.5)	80 (45.2)		
Lives with others	135 (23.6)	95 (24.1)	40 (22.6)		
Not stated	116 (20.3)	78 (19.8)	38 (21.5)		
**Total**	**571**	**394 (100)**	**177 (100)**	1.00 (0.04)	0.80
Accommodation type					
Secure housing	257 (78.1)	189 (79.4)	68 (74.7)		
Homeless	21 (6.4)	12 (5.0)	9 (9.9)		
Emergency/transitional housing	-	-	-		
Other	41 (12.5)	41 (17.2)	11 (12.1)		
**Total**	-	-	-	2.65 (0.09)	0.45

Variable	N	Goal achieved n (%)	Goal not achieved n (%)	χ^2 (V)	p-value
Need for interpreter services					
No interpreter needed	566 (99.6)	390 (99.5)	176 (100.0)		
Interpreter needed	-	-	-		
**Total**	-	-	-	0.90 (0.04)	0.34
Health conditions					
Mental illness	194 (40.1)	135 (40.8)	59 (38.6)		
Alcohol & other drugs	260 (53.7)	175 (52.9)	85 (55.6)		
Physical illness or injury	25 (5.2)	17 (5.1)	8 (5.2)		
Others	-	-	-		
**Total**	-	-	-	0.58 (0.04)	0.90
Mental health conditions					
F40 - F49	151 (44.4)	109 (45.8)	42 (41.2)		
F00 – F09	-	-	-		
F10 - F19	-	-	-		
F20 - F29	23 (6.8)	9 (3.8)	14 (13.7)		
F30 - F39	119 (35.0)	85 (35.7)	34 (33.3)		
F50 - F59	-	-	-		
F60 - F69	-	-	-		
F70 - F79	-	-	-		
F80 - F89	-	-	-		
F90 - F98	-	-	-		
F99	-	-	-		
**Total**	**340**	**238 (100)**	**102 (100)**	16.41 (0.22)	0.06
IRSD*					
< 50th percentile	237 (42.5)	157 (40.7)	80 (46.5)		
≥ 50th percentile	321 (57.5)	229 (59.3)	92 (53.5)		
**Total**	**558**	**386 (100)**	**172 (100)**	1.66 (0.06)	0.20

Variable	N	Goal achieved n (%)	Goal not achieved n (%)	χ^2 (V)	p-value
Crow distance category					
< 4 kms	156 (28.0)	104 (27.0)	52 (30.2)		
4-9 kms	194 (34.8)	128 (33.2)	66 (38.4)		
> 9 kms	207 (37.2)	153 (39.7)	54 (31.4)		
**Total**	**557**	**385 (100)**	**172 (100)**	3.56 (0.08)	0.17
Land distance category					
< 7 kms	158 (28.4)	97 (25.2)	61 (35.5)		
7-11 kms	192 (34.5)	135 (35.1)	57 (33.1)		
> 11 kms	207 (37.2)	153 (39.7)	54 (31.4)		
**Total**	**557**	**385 (100)**	**172 (100)**	6.78 (0.11)	0.03*
Travel time (minutes)					
< 9 mins	145 (26.0)	91 (23.6)	54 (31.4)		
9-15 mins	205 (36.8)	141 (36.6)	64 (37.2)		
> 15 mins	207 (37.2)	153 (39.7)	54 (31.4)		
**Total**	**557**	**385 (100)**	**172 (100)**	4.99 (0.10)	0.08

Notes. Univariate chi-square analyses using unique client-level data. Totals vary due to missing data; values are reported based on the number of non-missing observations (n) for each variable. Percentages for N are column percentages of the total sample. Percentages for outcome columns are calculated within outcome groups. Percentages may not equal 100 based on rounding.
*p<0.05; **p<0.01.

TSI = Torres Strait Islander. IRSD = Index of Relative Socio-economic Disadvantage. Other occupation = Home duties, Student, Other. V = Cramer's V effect size.

The codes listed under the 'mental health conditions' variable correspond to the groupings of mental and behavioural disorders as outlined in the International Classification of Diseases, 10th revision (ICD-10). They are as follows:

F00-F09	Organic, including symptomatic, mental disorders
F10-F19	Mental and behavioural disorders due to psychoactive substance use
F20-F29	Schizophrenia, schizotypal and delusional disorders
F30-F39	Mood [affective] disorders
F40-F49	Neurotic, stress-related and somatoform disorders
F50-F59	Behavioural syndromes associated with physiological disturbances & physical factors
F60-F69	Disorders of adult personality and behaviour
F70-F79	Mental retardation
F80-F89	Disorders of psychological development
F90-F98	Behavioural and emotional disorders with onset usually occurring in childhood and adolescence
F99	Unspecified mental disorder

Table S9 Client characteristics by treatment goal achievement status (episode-level data)

Variable	*N*	Goal achieved *n* (%)	Goal not achieved *n* (%)	OR (95% CI)	*p*-value
Country of birth					
Australia (ref)	543 (81.9)	368 (80.9)	175 (84.1)		
Asia	45 (6.8)	34 (7.5)	11 (5.3)	1.54 (0.71-3.35)	0.28
Oceania	32 (4.8)	25 (5.5)	7 (3.4)	1.76 (0.70-4.47)	0.23
Europe	-	-	-	1.83 (0.55-6.04)	0.32
Africa and others	24 (3.6)	13 (2.9)	11 (5.3)	0.54 (0.22-1.34)	0.18
**Total**	-	-	-		
Age category					
18-25 (ref)	144 (21.4)	95 (20.5)	49 (23.2)		
26-40	366 (54.3)	249 (53.8)	117 (55.5)	1.12 (0.71-1.75)	0.63
41 and above	164 (24.3)	119 (25.7)	45 (21.3)	1.40 (0.82-2.40)	0.22
**Total**	**674**	**463 (100)**	**211 (100)**		
Sex					
Male (ref)	553 (82.0)	379 (81.9)	174 (82.5)		
Female	121 (18.0)	84 (18.1)	37 (17.5)	1.04 (0.66-1.65)	0.85
**Total**	**674**	**463 (100)**	**211 (100)**		
Marital status					
Single (ref)	455 (67.5)	305 (65.9)	150 (71.1)		
De facto	79 (11.7)	60 (13.0)	19 (9.0)	1.61 (0.87-2.98)	0.13
Divorced	-	-	-	1.53 (0.44-5.29)	0.50
Married	30 (4.5)	22 (4.8)	8 (3.8)	1.37 (0.56-3.36)	0.50
Not known	69 (10.2)	47 (10.2)	22 (10.4)	1.05 (0.59-1.89)	0.86
Separated	25 (3.7)	17 (3.7)	8 (3.8)	1.04 (0.41-2.66)	0.93
**Total**	-	-	-		
Preferred language					
English (ref)	658 (97.6)	454 (98.1)	204 (96.7)		
Others	16 (2.4)	9 (1.9)	7 (3.3)	0.55 (0.19-1.66)	0.29
**Total**	**674**	**463 (100)**	**211 (100)**		

Variable	*N*	Goal achieved *n* (%)	Goal not achieved *n* (%)	OR (95% CI)	*p*-value
Aboriginal status					
Not Indigenous (ref)	635 (94.2)	440 (95.0)	195 (92.4)		
Aboriginal or TSI	32 (4.7)	18 (3.9)	14 (6.6)	0.54 (0.24-1.22)	0.14
Declined to answer	7 (1.0)	5 (1.1)	2 (0.9)	1.12 (0.19-6.51)	0.90
**Total**	**674**	**463 (100)**	**211 (100)**		
Employment status					
Unemployed (ref)	425 (74.0)	276 (69.7)	149 (83.7)		
Employed	136 (23.7)	110 (27.8)	26 (14.6)	2.44 (1.38-4.31)	0.002**
Other occupation	-	-	-	1.92 (0.46-8.04)	0.37
**Total**	-	-	-		
Refugee status					
Not a refugee or asylum seeker (ref)	640 (99.4)	443 (99.8)	197 (98.5)		
Refugee or asylum seeker	-	-	-	0.12 (0.01-1.63)	0.11
**Total**	-	-	-		
Carer status					
Does not have carer (ref)	34 (81.0)	20 (76.9)	14 (87.5)		
Has a carer	-	-	-	Insufficient data	
**Total**	-	-	-		
Living arrangement					
Lives alone (ref)	61 (9.1)	37 (8.0)	24 (11.4)		
Lives with family	314 (46.6)	220 (47.5)	94 (44.5)	1.53 (0.84-2.81)	0.17
Lives with others	164 (24.3)	114 (24.6)	50 (23.7)	1.49 (0.78-2.86)	0.23
Not stated	135 (20.0)	92 (19.9)	43 (20.4)	1.39 (0.72-2.71)	0.33
**Total**	**674**	**463 (100)**	**211 (100)**		
Accommodation type					
Secure housing (ref)	298 (75.8)	218 (78.1)	80 (70.2)		
Homeless	29 (7.4)	15 (5.4)	14 (12.3)	0.39 (0.18-0.85)	0.02*
Emergency/transitional housing	-	-	-	0.64 (0.18-2.25)	0.49
Other	55 (14.0)	39 (14.0)	16 (14.0)	0.90 (0.47-1.69)	0.73
**Total**	-	-	-		

**Variable**	***N***	**Goal achieved *n* (%)**	**Goal not achieved *n* (%)**	**OR (95% CI)**	***p*-value**
Need for interpreter					
No interpreter needed (ref)	669 (99.7)	458 (99.6)	211 (100.0)		
Interpreter needed	-	-	-	Insufficient data	
**Total**	-	-	-		
Health conditions					
Mental illness (ref)	231 (40.8)	161 (41.9)	70 (38.5)		
Alcohol & other drugs	300 (53.0)	198 (51.6)	102 (56.0)	0.84 (0.58-1.22)	0.37
Physical illness or injury	30 (5.3)	21 (5.5)	9 (4.9)	1.02 (0.44-2.33)	0.97
Others	-	-	-	1.74 (0.19-15.84)	0.62
**Total**	-	-	-		
Mental health conditions					
F40 - F49 (ref)	177 (45.0)	130 (46.9)	47 (40.5)		
F00 – F09	-	-	-	Insufficient data	
F10 - F19	-	-	-	4.34 (0.55-34.28)	0.16
F20 - F29	24 (6.1)	9 (3.2)	15 (12.9)	0.22 (0.09-0.53)	0.001**
F30 - F39	139 (35.4)	98 (35.4)	41 (35.3)	0.86 (0.53-1.42)	0.56
F50 - F59	177 (45.0)	130 (46.9)	47 (40.5)	Insufficient data	
F60 - F69	-	-	-	1.09 (0.28-4.18)	0.91
F70 - F79	-	-	-	Insufficient data	
F80 - F89	-	-	-	1.09 (0.11-10.69)	0.95
F90 - F98	-	-	-	1.09 (0.28-4.18)	0.91
F99	-	-	-	0.29 (0.08-1.12)	0.07
**Total**	**393**	**277 (100)**	**116 (100)**		
IRSD*					
< 50th percentile (ref)	283 (43.1)	186 (41.2)	97 (47.5)		
≥ 50th percentile	373 (56.9)	266 (58.8)	107 (52.5)	1.32 (0.92-1.89)	0.14
**Total**	**656**	**452 (100)**	**204 (100)**		

Variable	*N*	Goal achieved *n* (%)	Goal not achieved *n* (%)	OR (95% CI)	*p*-value
Crow distance category					
< 4 kms (ref)	188 (28.7)	125 (27.8)	63 (30.9)		
4-9 kms	228 (34.9)	157 (34.9)	71 (34.8)	1.12 (0.72-1.72)	0.62
> 9 kms	238 (36.4)	168 (37.3)	70 (34.3)	1.23 (0.79-1.90)	0.37
**Total**	**654**	**450 (100)**	**204 (100)**		
Land distance category					
< 7 kms (ref)	184 (28.1)	115 (25.6)	69 (33.8)		
7-11 kms	232 (35.5)	167 (37.1)	65 (31.9)	1.56 (1.00-2.43)	0.049*
> 11 kms	238 (36.4)	168 (37.3)	70 (34.3)	1.46 (0.94-2.28)	0.09
**Total**	**654**	**450 (100)**	**204 (100)**		
Travel time (minutes)					
< 9 mins (ref)	171 (26.1)	109 (24.2)	62 (30.4)		
9-15 mins	245 (37.5)	173 (38.4)	72 (35.3)	1.38 (0.89-2.14)	0.15
> 15 mins	238 (36.4)	168 (37.3)	70 (34.3)	1.39 (0.88-2.18)	0.16
**Total**	**654**	**450 (100)**	**204 (100)**		

Notes. Bivariate logistic regression analyses using episode-level data. Reference category listed first and marked with (ref) in the OR calculations. Totals vary due to missing data; values are reported based on the number of non-missing observations (*n*) for each variable. Percentages for N are column percentages of the total sample. Percentages for outcome columns are calculated within outcome groups. Percentages may not equal 100 based on rounding. * p<0.05; **p<0.01.

TSI = Torres Strait Islander. IRSD = Index of Relative Socio-economic Disadvantage. OR = Odds ratio. CI = Confidence interval. Other occupation = Home duties, Student, Other.

The codes listed under the 'mental health conditions' variable correspond to the groupings of mental and behavioural disorders as outlined in the International Classification of Diseases, 10th revision (ICD-10). They are as follows:

F00-F09	Organic, including symptomatic, mental disorders
F10-F19	Mental and behavioural disorders due to psychoactive substance use
F20-F29	Schizophrenia, schizotypal and delusional disorders
F30-F39	Mood [affective] disorders
F40-F49	Neurotic, stress-related and somatoform disorders
F50-F59	Behavioural syndromes associated with physiological disturbances & physical factors
F60-F69	Disorders of adult personality and behaviour
F70-F79	Mental retardation
F80-F89	Disorders of psychological development
F90-F98	Behavioural and emotional disorders with onset usually occurring in childhood and adolescence
F99	Unspecified mental disorder

Table S10 Treatment completion (discharge reason) status by group

Discharge reasons	Total *n* (%)	Exercise physiology *n* (%)			Speech pathology *n* (%)		
		Yes	No	Missing	Yes	No	Missing
Completed treatment	350 (31.5)	79 (22.6)	214 (61.4)	57 (16.3)	39 (11.1)	254 (72.6)	57 (16.3)
Did not engage	380 (34.2)	34 (9.0)	284 (74.7)	62 (16.3)	27 (7.1)	291 (76.6)	62 (16.3)
Became unavailable	222 (20.0)	15 (6.8)	150 (67.6)	57 (25.7)	11 (5.0)	154 (69.4)	57 (25.7)
No treatment required	47 (4.2)	0 (0.0)	38 (80.9)	9 (19.2)	0 (0.0)	38 (80.9)	9 (19.2)
Eliminated	102 (9.2)	2 (2.0)	26 (25.5)	74 (72.6)	1 (1.0)	27 (26.5)	74 (72.6)
Missing data	10 (0.9)	0 (0.0)	8 (80.0)	2 (20.0)	1 (10.0)	7 (70.0)	2 (20.0)
**Total**	**1111 (100.0)**	**130 (11.7)**	**720 (64.8)**	**261 (23.5)**	**79 (7.1)**	**771 (69.4)**	**261 (23.5)**

Note. Percentages may not equal 100 based on rounding.

Table S11 Treatment goal achievement status by group

Treatment goal	Total *n* (%)	Exercise physiology *n* (%)			Speech pathology *n* (%)		
		Yes	No	Missing	Yes	No	Missing
Not achieved	177 (15.9)	11 (6.2)	160 (90.4)	6 (3.4)	7 (4.0)	164 (92.7)	6 (3.4)
Achieved	252 (22.7)	73 (29.0)	174 (69.1)	5 (2.0)	38 (15.1)	209 (82.9)	5 (2.0)
Partially achieved	142 (12.8)	29 (20.4)	110 (77.5)	3 (2.1)	18 (12.7)	121 (85.2)	3 (2.1)
Other	99 (8.9)	0 (0.0)	96 (97.0)	3 (3.0)	0 (0.0)	96 (97.0)	3 (3.0)
Missing data	441 (39.7)	17 (3.9)	180 (40.8)	244 (55.3)	16 (3.6)	181 (41.0)	244 (55.3)
**Total**	**1111 (100.0)**	**130 (11.7)**	**720 (64.8)**	**261 (23.5)**	**79 (7.1)**	**771 (69.4)**	**261 (23.5)**

Note. Percentages may not equal 100 based on rounding.

Table S12 Client characteristics by exercise physiology participation status (client-level data)

Variable	N	Participation in EP		χ^2 (*V*)	*p*-value
		No - *n* (%)	Yes - *n* (%)		
Country of birth					
Australia	683 (81.4)	577 (81.0)	106 (83.5)		
Asia	62 (7.4)	54 (7.6)	8 (6.3)		
Oceania	35 (4.2)	30 (4.2)	5 (3.9)		
Europe	26 (3.1)	21 (3.0)	5 (3.9)		
Africa and others	-	-	-		
**Total**	-	-	-	1.62 (0.04)	0.81
Age category					
18-25	170 (20.0)	146 (20.3)	24 (18.5)		
26-40	465 (54.8)	395 (54.9)	70 (53.8)		
41 and above	214 (25.2)	178 (24.8)	36 (27.7)		
**Total**	**849**	**719 (100)**	**130 (100)**	0.59 (0.03)	0.75
Sex					
Male	697 (82.0)	583 (81.0)	114 (87.7)		
Female	153 (18.0)	137 (19.0)	16 (12.3)		
**Total**	**850**	**720 (100)**	**130 (100)**	3.37 (0.06)	0.07
Marital status					
Single	581 (68.4)	496 (68.9)	85 (65.4)		
De facto	90 (10.6)	67 (9.3)	23 (17.7)		
Divorced	23 (2.7)	16 (2.2)	7 (5.4)		
Married	-	-	-		
Not known	86 (10.1)	78 (10.8)	8 (6.2)		
Separated	-	-	-		
**Total**	**850**	**720 (100)**	**130 (100)**	15.66 (0.14)	0.008
Preferred language					
English	823 (97.1)	696 (96.8)	127 (98.4)		
Others	-	-	-		
**Total**	-	-	-	1.04 (0.04)	0.31

Variable	N	Participation in EP		χ^2 (V)	p-value
		No - n (%)	Yes - n (%)		
Aboriginal status					
Not Indigenous	790 (92.9)	666 (92.5)	124 (95.4)		
Aboriginal or TSI	50 (5.9)	44 (6.1)	6 (4.6)		
Declined to answer	10 (1.2)	10 (1.4)	0 (0.0)		
**Total**	**850**	**720 (100)**	**130 (100)**	2.32 (0.05)	0.31
Employment status					
Unemployed	533 (74.4)	446 (74.1)	87 (76.3)		
Employed	167 (23.3)	144 (23.9)	23 (20.2)		
Other occupation	-	-	-		
**Total**	-	-	-	1.63 (0.05)	0.44
Refugee status					
Not a refugee or asylum seeker	808 (99.6)	686 (99.6)	122 (100.0)		
Refugee or asylum seeker	-	-	-		
**Total**	-	-	-	0.53 (0.03)	0.47
Carer status					
No carer	-	-	-		
Has a carer	-	-	-		
**Total**	-	-	-	0.55 (0.11)	0.46
Living arrangement					
Lives alone	77 (9.1)	68 (9.4)	9 (6.9)		
Lives with family	382 (44.9)	319 (44.3)	63 (48.5)		
Lives with others	204 (24.0)	173 (24.0)	31 (23.8)		
Not stated	187 (22.0)	160 (22.2)	27 (20.8)		
**Total**	**850**	**720 (100)**	**130 (100)**	1.30 (0.04)	0.73
Accommodation type					
Secure housing	382 (77.0)	316 (76.5)	66 (79.5)		
Homeless	-	-	-		
Emergency/transitional housing	-	-	-		
Other	60 (12.1)	46 (11.1)	14 (16.9)		
**Total**	**496**	**413 (100)**	**83 (100)**	7.26 (0.12)	0.06

Variable	N	Participation in EP		χ^2 (V)	p-value
		No n (%)	Yes n (%)		
Need for interpreter					
No interpreter needed	839 (99.1)	711 (99.0)	128 (99.2)		
Interpreter needed	-	-	-		
**Total**	-	-	-	0.05 (0.01)	0.83
Health conditions					
Mental illness	256 (35.8)	214 (35.3)	42 (38.9)		
Alcohol & other drugs	417 (58.3)	363 (59.8)	54 (50.0)		
Physical illness or injury	35 (4.9)	25 (4.1)	10 (9.3)		
Others	-	-	-		
**Total**	-	-	-	7.79 (0.10)	0.05
Mental health conditions					
F40 - F49	212 (42.8)	179 (42.1)	33 (47.1)		
F00 – F09	-	-	-		
F10 - F19	-	-	-		
F20 - F29	-	-	-		
F30 - F39	181 (36.6)	156 (36.7)	25 (35.7)		
F50 - F59	-	-	-		
F60 - F69	-	-	-		
F70 - F79	-	-	-		
F80 - F89	-	-	-		
F90 - F98	-	-	-		
F99	-	-	-		
**Total**	**495**	**425 (100)**	**70 (100)**	3.60 (0.09)	0.96
IRSD*					
< 50th percentile	362 (43.3)	298 (42.1)	64 (50.0)		
≥ 50th percentile	474 (56.7)	410 (57.9)	64 (50.0)		
**Total**	**836**	**708 (100)**	**128 (100)**	2.76 (0.06)	0.10

Variable	N	Participation in EP		χ^2 (V)	p-value
		No n (%)	Yes n (%)		
Crow distance category					
< 4 kms	234 (28.1)	189 (26.7)	45 (35.7)		
4-9 kms	307 (36.9)	263 (37.2)	44 (34.9)		
> 9 kms	292 (35.1)	255 (36.1)	37 (29.4)		
**Total**	**833**	**707 (100)**	**126 (100)**	4.59 (0.07)	0.10
Land distance category					
< 7 kms	243 (29.2)	200 (28.3)	43 (34.1)		
7-11 kms	298 (35.8)	252 (35.6)	46 (36.5)		
> 11 kms	292 (35.1)	255 (36.1)	37 (29.4)		
**Total**	**833**	**707 (100)**	**126 (100)**	2.64 (0.06)	0.27
Travel time (minutes)					
< 9 mins	219 (26.3)	177 (25.0)	42 (33.3)		
9-15 mins	322 (38.7)	275 (38.9)	47 (37.3)		
> 15 mins	292 (35.1)	255 (36.1)	37 (29.4)		
**Total**	**833**	**707 (100)**	**126 (100)**	4.24 (0.07)	0.12

Notes. Univariate chi-square analyses using unique client-level data. Totals vary due to missing data; values are reported based on the number of non-missing observations (n) for each variable. Percentages for N are column percentages of the total sample. Percentages for outcome columns are calculated within outcome groups. Percentages may not equal 100 based on rounding. *p<0.05; **p<0.01.

TSI = Torres Strait Islander. IRSD = Index of Relative Socio-economic Disadvantage. Other occupation = Home duties, Student, Other. V = Cramer's V effect size.

The codes listed under the 'mental health conditions' variable correspond to the groupings of mental and behavioural disorders as outlined in the International Classification of Diseases, 10th revision (ICD-10). They are as follows:

F00-F09	Organic, including symptomatic, mental disorders
F10-F19	Mental and behavioural disorders due to psychoactive substance use
F20-F29	Schizophrenia, schizotypal and delusional disorders
F30-F39	Mood [affective] disorders
F40-F49	Neurotic, stress-related and somatoform disorders
F50-F59	Behavioural syndromes associated with physiological disturbances & physical factors
F60-F69	Disorders of adult personality and behaviour
F70-F79	Mental retardation
F80-F89	Disorders of psychological development
F90-F98	Behavioural and emotional disorders with onset usually occurring in childhood and adolescence
F99	Unspecified mental disorder

Table S13 Client characteristics by exercise physiology participation status (episode-level data)

Variable	*N*	Participation in EP		OR (95% CI)	*p*-value
		No - *n* (%)	Yes - *n* (%)		
Country of birth					
Australia (ref)	808 (81.7)	689 (81.5)	119 (82.6)		
Asia	74 (7.5)	64 (7.6)	10 (6.9)	0.91 (0.45-1.81)	0.78
Oceania	43 (4.3)	36 (4.3)	7 (4.9)	1.13 (0.49-2.59)	0.78
Europe	29 (2.9)	24 (2.8)	5 (3.5)	1.21 (0.45-3.22)	0.71
Africa and others	-	-	-	0.54 (0.16-1.80)	0.32
**Total**	-	-	-		
Age category					
18-25 (ref)	208 (20.8)	178 (20.8)	30 (20.4)		
26-40	555 (55.4)	480 (56.1)	75 (51.0)	0.93 (0.59-1.46)	0.75
41 and above	239 (23.9)	197 (23.0)	42 (28.6)	1.27 (0.76-2.11)	0.37
**Total**	**1002**	**855 (100)**	**147 (100)**		
Sex					
Male (ref)	825 (82.3)	699 (81.7)	126 (85.7)		
Female	178 (17.7)	157 (18.3)	21 (14.3)	0.74 (0.45-1.22)	0.24
**Total**	**1003**	**856 (100)**	**147 (100)**		
Marital status					
Single (ref)	695 (69.3)	598 (69.9)	97 (66.0)		
De facto	107 (10.7)	83 (9.7)	24 (16.3)	1.78 (1.08-2.95)	0.02*
Divorced	24 (2.4)	17 (2.0)	7 (4.8)	2.54 (1.03-6.28)	0.04*
Married	52 (5.2)	47 (5.5)	5 (3.4)	0.66 (0.26-1.69)	0.38
Not known	94 (9.4)	86 (10.0)	8 (5.4)	0.57 (0.27-1.22)	0.15
Separated	31 (3.1)	25 (2.9)	6 (4.1)	1.48 (0.59-3.70)	0.40
**Total**	**1003**	**856 (100)**	**147 (100)**		
Preferred language					
English (ref)	973 (97.2)	829 (97.0)	144 (98.6)		
Others	-	-	-	0.44 (0.10-1.89)	0.27
**Total**	-	-	-		

Variable	N	Participation in EP		OR (95% CI)	p-value
		No - n (%)	Yes - n (%)		
Aboriginal status					
Not Indigenous (ref)	932 (92.9)	794 (92.8)	138 (93.9)		
Aboriginal or TSI*	60 (6.0)	51 (6.0)	9 (6.1)	1.02 (0.49-2.11)	0.97
Declined to answer	11 (1.1)	11 (1.3)	0 (0.0)	Insufficient data	
**Total**	**1003**	**856 (100)**	**147 (100)**		
Employment status					
Unemployed (ref)	641 (75.7)	545 (75.6)	96 (76.2)		
Employed	187 (22.1)	161 (22.3)	26 (20.6)	0.92 (0.57-1.46)	0.72
Other occupation	-	-	-	1.51 (0.49-4.66)	0.36
**Total**	-	-	-		
Refugee status					
Not a refugee or asylum seeker (ref)	957 (99.6)	819 (99.5)	138 (100.0)		
Refugee or asylum seeker	-	-	-	Insufficient data	
**Total**	-	-	-		
Carer status					
No carer (ref)	-	-	-		
Has a carer	-	-	-	Insufficient data	
**Total**	-	-	-		
Living arrangement					
Lives alone (ref)	89 (8.9)	78 (9.1)	11 (7.5)		
Lives with family	455 (45.4)	386 (45.1)	69 (46.9)	1.27 (0.64-2.51)	0.50
Lives with others	246 (24.5)	209 (24.4)	37 (25.2)	1.26 (0.61-2.58)	0.54
Not stated	213 (21.2)	183 (21.4)	30 (20.4)	1.16 (0.56-2.44)	0.69
**Total**	**1003**	**856 (100)**	**147 (100)**		
Accommodation type					
Secure housing (ref)	451 (76.3)	377 (76.2)	74 (77.1)		
Homeless	-	-	-	0.46 (0.16-1.33)	0.15
Emergency/transitional housing	-	-	-	0.34 (0.04-2.61)	0.30
Other	76 (12.9)	59 (11.9)	17 (17.7)	1.47 (0.81-2.66)	0.21
**Total**	**591**	**495 (100)**	**96 (100)**		

Variable	*N*	Participation in EP		OR (95% CI)	*p*-value
		No *n* (%)	Yes *n* (%)		
Need for interpreter					
No interpreter needed (ref)	990 (99.2)	846 (99.2)	144 (99.3)		
Interpreter needed	-	-	-	0.84 (0.10-6.87)	0.87
**Total**	-	-	-		
Health conditions					
Mental illness (ref)	307 (36.6)	257 (35.7)	50 (41.7)		
Alcohol & other drugs	485 (57.8)	427 (59.4)	58 (48.3)	0.70 (0.46-1.05)	0.09
Physical illness or injury	40 (4.8)	30 (4.2)	10 (8.3)	1.71 (0.79-3.73)	0.17
Others	-	-	-	2.06 (0.39-10.90)	0.40
**Total**	-	-	-		
Mental health conditions					
F40 - F49 (ref)	250 (43.9)	214 (43.5)	36 (46.8)		
F00 – F09	-	-	-	Insufficient data	
F10 - F19	-	-	-	0.92 (0.20-4.22)	0.91
F20 - F29	-	-	-	0.53 (0.15-1.80)	0.31
F30 - F39	206 (36.2)	178 (36.2)	28 (36.4)	0.94 (0.55-1.59)	0.81
F50 - F59	-	-	-	Insufficient data	
F60 - F69	-	-	-	1.12 (0.31-4.02)	0.87
F70 - F79	-	-	-	Insufficient data	
F80 - F89	-	-	-	Insufficient data	
F90 - F98	-	-	-	0.66 (0.15-2.97)	0.59
F99	-	-	-	1.62 (0.43-6.10)	0.48
**Total**	**569**	**492 (100)**	**77 (100)**		
IRSD*					
< 50th percentile (ref)	435 (44.3)	363 (43.2)	72 (50.7)		
≥ 50th percentile	548 (55.7)	478 (56.8)	70 (49.3)	0.74 (0.52-1.05)	0.10
**Total**	**983**	**841 (100)**	**142 (100)**		

Variable	*N*	Participation in EP		OR (95% CI)	*p*-value
		No *n* (%)	Yes *n* (%)		
Crow distance category					
< 4 kms (ref)	281 (28.7)	231 (27.5)	50 (35.7)		
4-9 kms	361 (36.9)	315 (37.5)	46 (32.9)	0.68 (0.44-1.04)	0.08
> 9 kms	337 (34.4)	293 (34.9)	44 (31.4)	0.69 (0.45-1.08)	0.10
**Total**	**979**	**839 (100)**	**140 (100)**		
Land distance category					
< 7 kms (ref)	287 (29.3)	239 (28.5)	48 (34.3)		
7-11 kms	355 (36.3)	307 (36.6)	48 (34.3)	0.78 (0.50-1.20)	0.26
> 11 kms	337 (34.4)	293 (34.9)	44 (31.4)	0.75 (0.48-1.17)	0.20
**Total**	**979**	**839 (100)**	**140 (100)**		
Travel time (minutes)					
< 9 mins (ref)	262 (26.8)	215 (25.6)	47 (33.6)		
9-15 mins	380 (38.8)	331 (39.5)	49 (35.0)	0.68 (0.44-1.05)	0.08
> 15 mins	337 (34.4)	293 (34.9)	44 (31.4)	0.69 (0.44-1.07)	0.10
**Total**	**979**	**839 (100)**	**140 (100)**		

Notes. Bivariate logistic regression analyses using episode-level data. Reference category listed first and marked with (ref) in the OR calculations. Totals vary due to missing data; values are reported based on the number of non-missing observations (*n*) for each variable. Percentages for N are column percentages of the total sample. Percentages for outcome columns are calculated within outcome groups. Percentages may not equal 100 based on rounding. * p<0.05.

TSI = Torres Strait Islander. IRSD = Index of Relative Socio-economic Disadvantage. OR = Odds ratio. CI = Confidence interval. Other occupation = Home duties, Student, Other.

The codes listed under the 'mental health conditions' variable correspond to the groupings of mental and behavioural disorders as outlined in the International Classification of Diseases, 10th revision (ICD-10). They are as follows:

F00-F09	Organic, including symptomatic, mental disorders
F10-F19	Mental and behavioural disorders due to psychoactive substance use
F20-F29	Schizophrenia, schizotypal and delusional disorders
F30-F39	Mood [affective] disorders
F40-F49	Neurotic, stress-related and somatoform disorders
F50-F59	Behavioural syndromes associated with physiological disturbances & physical factors
F60-F69	Disorders of adult personality and behaviour
F70-F79	Mental retardation
F80-F89	Disorders of psychological development
F90-F98	Behavioural and emotional disorders with onset usually occurring in childhood and adolescence
F99	Unspecified mental disorder

Table S14 Client characteristics by speech pathology participation status (client-level data)

Variable	N	Participation in SP		χ^2 (*V*)	*p*-value
		No – *n* (%)	Yes – *n* (%)		
Country of birth					
Australia	683 (81.4)	613 (80.7)	70 (88.6)		
Asia	-	-	-		
Oceania	-	-	-		
Europe	-	-	-		
Africa and others	-	-	-		
**Total**	**839**	**760 (100)**	**79 (100)**	5.09 (0.08)	0.28
Age category					
18-25	170 (20.0)	158 (20.5)	12 (15.2)		
26-40	465 (54.8)	423 (54.9)	42 (53.2)		
41 and above	214 (25.2)	189 (24.5)	25 (31.6)		
**Total**	**849**	**770 (100)**	**79 (100)**	2.49 (0.05)	0.29
Sex					
Male	697 (82.0)	629 (81.6)	68 (86.1)		
Female	153 (18.0)	142 (18.4)	11 (13.9)		
**Total**	**850**	**771 (100)**	**79 (100)**	0.98 (0.03)	0.32
Marital status					
Single	581 (68.4)	529 (68.6)	52 (65.8)		
De facto	90 (10.6)	76 (9.9)	14 (17.7)		
Divorced	-	-	-		
Married	-	-	-		
Not known	-	-	-		
Separated	-	-	-		
**Total**	**850**	**771 (100)**	**79 (100)**	9.88 (0.11)	0.08
Preferred language					
English	823 (97.1)	749 (97.3)	74 (94.9)		
Others	-	-	-		
**Total**	-	-	-	1.43 (0.04)	0.23

Variable	N	Participation in SP		χ^2 (V)	p-value
		No – n (%)	Yes – n (%)		
Aboriginal status					
Not Indigenous	790 (92.9)	716 (92.9)	74 (93.7)		
Aboriginal or TSI	-	-	-		
Declined to answer	-	-	-		
**Total**	**850**	**771 (100)**	**79 (100)**	0.11 (0.01)	0.95
Employment status					
Unemployed	533 (74.4)	489 (75.1)	44 (67.7)		
Employed	167 (23.3)	150 (23.0)	17 (26.2)		
Other occupation	-	-	-		
**Total**	-	-	-	5.60 (0.09)	0.06
Refugee status					
Not a refugee or asylum seeker	808 (99.6)	733 (99.6)	75 (100.0)		
Refugee or asylum seeker	-	-	-		
**Total**	-	-	-	0.31 (0.02)	0.58
Carer status					
No carer	38 (79.2)	38 (79.2)	No data		
Has a carer	10 (20.8)	10 (20.8)	No data		
**Total**	**48**	**48 (100.0)**	No data	Insufficient data	
Living arrangement					
Lives alone	77 (9.1)	68 (8.8)	9 (11.4)		
Lives with family	382 (44.9)	354 (45.9)	28 (35.4)		
Lives with others	204 (24.0)	184 (23.9)	20 (25.3)		
Not stated	187 (22.0)	165 (21.4)	22 (27.8)		
**Total**	**850**	**771 (100)**	**79 (100)**	3.69 (0.07)	0.30
Accommodation type					
Secure housing	382 (77.0)	350 (77.3)	32 (74.4)		
Homeless	-	-	-		
Emergency/transitional housing	-	-	-		
Other	60 (12.1)	51 (11.3)	9 (20.9)		
**Total**	**496**	**453 (100)**	**43 (100)**	5.12 (0.10)	0.16

Variable	*N*	Participation in SP		χ^2 (*V*)	*p*-value
		No – *n* (%)	Yes – *n* (%)		
Need for interpreter services					
No interpreter needed	839 (99.1)	760 (99.0)	79 (100.0)		
Interpreter needed	-	-	-		
**Total**	-	-	-	0.83 (0.03)	0.36
Health conditions					
Mental illness	256 (35.8)	218 (33.7)	38 (55.9)		
Alcohol & other drugs	417 (58.3)	393 (60.7)	24 (35.3)		
Physical illness or injury	35 (4.9)	30 (4.6)	5 (7.4)		
Others	-	-	-		
**Total**	-	-	-	16.41 (0.15)	0.001**
Mental health conditions					
F40 - F49	212 (42.8)	194 (42.7)	18 (43.9)		
F00 – F09	-	-	-		
F10 - F19	-	-	-		
F20 - F29	-	-	-		
F30 - F39	181 (36.6)	166 (36.6)	15 (36.6)		
F50 - F59	-	-	-		
F60 - F69	-	-	-		
F70 - F79	-	-	-		
F80 - F89	-	-	-		
F90 - F98	-	-	-		
F99	-	-	-		
**Total**	**495**	**454 (100)**	**41 (100)**	16.26 (0.18)	0.09
IRSD*					
< 50th percentile	362 (43.3)	327 (43.2)	35 (44.3)		
≥ 50th percentile	474 (56.7)	430 (56.8)	44 (55.7)		
**Total**	**836**	**757 (100)**	**79 (100)**	0.04 (0.01)	0.85

Variable	*N*	Participation in SP		χ^2 (*V*)	*p*-value
		No *n* (%)	Yes *n* (%)		
Crow distance category					
< 4 kms	234 (28.1)	214 (28.4)	20 (25.3)		
4-9 kms	307 (36.9)	274 (36.3)	33 (41.8)		
> 9 kms	292 (35.1)	266 (35.3)	26 (32.9)		
**Total**	**833**	**754 (100)**	**79 (100)**	0.93 (0.03)	0.63
Land distance category					
< 7 kms	243 (29.2)	221 (29.3)	22 (27.8)		
7-11 kms	298 (35.8)	267 (35.4)	31 (39.2)		
> 11 kms	292 (35.1)	266 (35.3)	26 (32.9)		
**Total**	**833**	**754 (100)**	**79 (100)**	0.46 (0.02)	0.80
Travel time (minutes)					
< 9 mins	219 (26.3)	198 (26.3)	21 (26.6)		
9-15 mins	322 (38.7)	290 (38.5)	32 (40.5)		
> 15 mins	292 (35.1)	266 (35.3)	26 (32.9)		
**Total**	**833**	**754 (100)**	**79 (100)**	0.20 (0.02)	0.91

Notes. Univariate chi-square analyses using unique client-level data. Totals vary due to missing data; values are reported based on the number of non-missing observations (*n*) for each variable. Percentages for *N* are column percentages of the total sample. Percentages for outcome columns are calculated within outcome groups. Percentages may not equal 100 based on rounding. *p<0.05; **p<0.01.

TSI = Torres Strait Islander. IRSD = Index of Relative Socio-economic Disadvantage. Other occupation = Home duties, Student, Other. *V* = Cramer's V effect size.

The codes listed under the 'mental health conditions' variable correspond to the groupings of mental and behavioural disorders as outlined in the International Classification of Diseases, 10th revision (ICD-10). They are as follows:

F00-F09 Organic, including symptomatic, mental disorders
F10-F19 Mental and behavioural disorders due to psychoactive substance use
F20-F29 Schizophrenia, schizotypal and delusional disorders
F30-F39 Mood [affective] disorders
F40-F49 Neurotic, stress-related and somatoform disorders
F50-F59 Behavioural syndromes associated with physiological disturbances & physical factors
F60-F69 Disorders of adult personality and behaviour
F70-F79 Mental retardation
F80-F89 Disorders of psychological development
F90-F98 Behavioural and emotional disorders with onset usually occurring in childhood and adolescence
F99 Unspecified mental disorder

Table S15 Client characteristics by speech pathology participation status (episode-level data)

Variable	*N*	Participation in SP		OR (95% CI)	*p*-value
		No – *n* (%)	Yes – *n* (%)		
Country of birth					
Australia (ref)	808 (81.7)	728 (81.1)	80 (87.9)		
Asia	-	-	-	0.25 (0.06-1.05)	0.06
Oceania	-	-	-	0.68 (0.21-2.26)	0.53
Europe	-	-	-	1.90 (0.70-5.11)	0.21
Africa and others	-	-	-	0.27 (0.04-1.98)	0.20
**Total**	**989**	**898 (100)**	**91 (100)**		
Age category					
18-25 (ref)	208 (20.8)	193 (21.2)	15 (16.5)		
26-40	555 (55.4)	510 (56.0)	45 (49.5)	1.14 (0.62-2.08)	0.68
41 and above	239 (23.9)	208 (22.8)	31 (34.1)	1.92 (1.00-3.66)	0.049*
**Total**	**1002**	**911 (100)**	**91 (100)**		
Sex					
Male (ref)	825 (82.3)	747 (81.9)	78 (85.7)		
Female	178 (17.7)	165 (18.1)	13 (14.3)	0.76 (0.41-1.39)	0.37
**Total**	**1003**	**912 (100)**	**91 (100)**		
Marital status					
Single (ref)	695 (69.3)	633 (69.4)	62 (68.1)		
De facto	107 (10.7)	92 (10.1)	15 (16.5)	1.67 (0.91-3.05)	0.10
Divorced	-	-	-	2.04 (0.68-6.16)	0.21
Married	-	-	-	0.41 (0.10-1.72)	0.22
Not known	-	-	-	0.45 (0.16-1.28)	0.14
Separated	-	-	-	1.51 (0.51-4.46)	0.45
**Total**	**1003**	**912 (100)**	**91 (100)**		
Preferred language					
English (ref)	973 (97.2)	887 (97.4)	86 (95.6)		
Others	-	-	-	1.72 (0.58-5.07)	0.33
**Total**	-	-	-		

Variable	*N*	Participation in SP		OR (95% CI)	*p*-value
		No – *n* (%)	Yes – *n* (%)		
Aboriginal status					
Not Indigenous (ref)	932 (92.9)	846 (92.8)	86 (94.5)		
Aboriginal or TSI	-	-	-	0.70 (0.25-1.99)	0.51
Declined to answer	-	-	-	0.98 (0.12-7.78)	0.99
**Total**	**1003**	**912 (100)**	**91 (100)**		
Employment status					
Unemployed (ref)	641 (75.7)	592 (76.6)	49 (66.2)		
Employed	187 (22.1)	166 (21.5)	21 (28.4)	1.52 (0.89-2.62)	0.12
Other occupation	-	-	-	3.22 (1.03-10.08)	0.04*
**Total**	-	-	-		
Refugee status					
Not a refugee or asylum seeker (ref)	957 (99.6)	871 (99.5)	86 (100.0)		
Refugee or asylum seeker	-	-	-	Insufficient data	
**Total**	-	-	-		
Carer status					
No carer	46 (79.3)	46 (79.3)	No data		
Has a carer	12 (20.7)	12 (20.7)	No data	Insufficient data	
**Total**	**58**	**58 (100)**	No data		
Living arrangement					
Lives alone (ref)	89 (8.9)	80 (8.8)	9 (9.9)		
Lives with family	455 (45.4)	420 (46.1)	35 (38.5)	0.74 (0.34-1.60)	0.45
Lives with others	246 (24.5)	225 (24.7)	21 (23.1)	0.83 (0.37-1.89)	0.66
Not stated	213 (21.2)	187 (20.5)	26 (28.6)	1.24 (0.55-2.76)	0.61
**Total**	**1003**	**912 (100)**	**91 (100)**		
Accommodation type					
Secure housing (ref)	451 (76.3)	416 (76.8)	35 (71.4)		
Homeless	-	-	-	0.79 (0.23-2.68)	0.71
Emergency/transitional housing	-	-	-	Insufficient data	
Other	76 (12.9)	65 (12.0)	11 (22.4)	2.01 (0.97-4.15)	0.06
**Total**	**591**	**542 (100)**	**49 (100)**		

Variable	*N*	Participation in SP		OR (95% CI)	*p*-value
		No – *n* (%)	Yes – *n* (%)		
Need for interpreter services					
No interpreter needed (ref)	990 (99.2)	900 (99.1)	90 (100.0)		
Interpreter needed	8 (0.8)	8 (0.9)	0 (0.0)	Insufficient data	
**Total**	**998**	**908 (100)**	**90 (100)**		
Health conditions					
Mental illness (ref)	307 (36.6)	261 (34.3)	46 (58.2)		
Alcohol & other drugs	485 (57.8)	458 (60.3)	27 (34.2)	0.33 (0.20-0.55)	0.000***
Physical illness or injury	40 (4.8)	35 (4.6)	5 (6.3)	0.81 (0.30-2.18)	0.68
Others	-	-	-	0.95 (0.11-8.04)	0.96
**Total**	-	-	-		
Mental health conditions					
F40 - F49 (ref)	250 (43.9)	228 (43.7)	22 (46.8)		
F00 – F09	-	-	-	Insufficient data	
F10 - F19	-	-	-	1.59 (0.34-7.53)	0.56
F20 - F29	-	-	-	0.59 (0.13-2.63)	0.49
F30 - F39	206 (36.2)	189 (36.2)	17 (36.2)	0.93 (0.48-1.81)	0.84
F50 - F59	-	-	-	Insufficient data	
F60 - F69	-	-	-	0.58 (0.07-4.52)	0.60
F70 - F79	-	-	-	Insufficient data	
F80 - F89	-	-	-	3.46 (0.35-34.63)	0.29
F90 - F98	-	-	-	Insufficient data	
F99	-	-	-	0.80 (0.10-6.38)	0.83
**Total**	**569**	**522 (100)**	**47 (100)**		
IRSD*					
< 50th percentile (ref)	435 (44.3)	395 (44.3)	40 (44.0)		
≥ 50th percentile	548 (55.7)	497 (55.7)	51 (56.0)	1.01 (0.66-1.57)	0.95
**Total**	**983**	**892 (100)**	**91 (100)**		

Variable	N	Participation in SP		OR (95% CI)	*p*-value
		No – *n* (%)	Yes – *n* (%)		
Crow distance category					
< 4 kms (ref)	281 (28.7)	259 (29.2)	22 (24.2)		
4-9 kms	361 (36.9)	322 (36.3)	39 (42.9)	1.43 (0.83-2.47)	0.20
> 9 kms	337 (34.4)	307 (34.6)	30 (33.0)	1.15 (0.65-2.04)	0.63
**Total**	**979**	**888 (100)**	**91 (100)**		
Land distance category					
< 7 kms (ref)	287 (29.3)	260 (29.3)	27 (29.7)		
7-11 kms	355 (36.3)	321 (36.1)	34 (37.4)	1.02 (0.60-1.74)	0.94
> 11 kms	337 (34.4)	307 (34.6)	30 (33.0)	0.94 (0.55-1.62)	0.83
**Total**	**979**	**888 (100)**	**91 (100)**		
Travel time (minutes)					
< 9 mins (ref)	262 (26.8)	236 (26.6)	26 (28.6)		
9-15 mins	380 (38.8)	345 (38.9)	35 (38.5)	0.92 (0.54-1.57)	0.76
> 15 mins	337 (34.4)	307 (34.6)	30 (33.0)	0.89 (0.51-1.54)	0.67
**Total**	**979**	**888 (100)**	**91 (100)**		

Notes. Bivariate logistic regression analyses using episode-level data. Reference category listed first and marked with (ref) in the OR calculations. Totals vary due to missing data; values are reported based on the number of non-missing observations (*n*) for each variable. Percentages for N are column percentages of the total sample. Percentages for outcome columns are calculated within outcome groups. Percentages may not equal 100 based on rounding. * p<0.05; ***p<0.001.

TSI = Torres Strait Islander. IRSD = Index of Relative Socio-economic Disadvantage. OR = Odds ratio. CI = Confidence interval. Other occupation = Home duties, Student, Other.

The codes listed under the 'mental health conditions' variable correspond to the groupings of mental and behavioural disorders as outlined in the International Classification of Diseases, 10th revision (ICD-10). They are as follows:

F00-F09	Organic, including symptomatic, mental disorders
F10-F19	Mental and behavioural disorders due to psychoactive substance use
F20-F29	Schizophrenia, schizotypal and delusional disorders
F30-F39	Mood [affective] disorders
F40-F49	Neurotic, stress-related and somatoform disorders
F50-F59	Behavioural syndromes associated with physiological disturbances & physical factors
F60-F69	Disorders of adult personality and behaviour
F70-F79	Mental retardation
F80-F89	Disorders of psychological development
F90-F98	Behavioural and emotional disorders with onset usually occurring in childhood and adolescence
F99	Unspecified mental disorder

Table S16 Conditional imputation models for the analysis of discharge reasons

Imputed variables	Model	Variable in the model
Discharge reason (completed treatment)	Logistic regression	Exercise physiology, speech pathology, age, sex, country of birth, IRSD decile scores, marital status, language, Aboriginal status, employment, living arrangement, health conditions, and Crow distance
IRSD decile	Linear regression	Discharge reasons, exercise physiology, Speech Pathology, age, sex, country of birth marital status, language, Aboriginal status, employment, living arrangement, health conditions, and Crow distance
Crow distance	Linear regression	Discharge reasons, exercise physiology, Speech Pathology, age, sex, country of birth, IRSD decile scores, marital status, language, Aboriginal status, employment, living arrangement, health conditions
Employment status	Multinomial logistic regression	Discharge reasons, exercise physiology, Speech Pathology, age, sex, country of birth, IRSD decile scores, marital status, language, Aboriginal status, living arrangement, health conditions, and Crow distance
Health conditions	Multinomial logistic regression	Discharge reasons, exercise physiology, Speech Pathology, age, sex, country of birth, IRSD decile scores, marital status, language, Aboriginal status, living arrangement, employment status, and Crow distance

Table S17 Results from imputation (outcome: completed treatment)

Imputed variables	Complete	Incomplete	Imputed	Total
Discharge reason	723	9	9	732
IRSD decile	720	12	12	732
Crow distance	719	13	13	732
Employment status	619	113	113	732
Health conditions	609	123	123	732

Table S18 Conditional imputation models for the analysis of treatment goals

Imputed variables	Model	Variable in the model
Treatment goal	Logistic regression	Exercise physiology, Speech Pathology, age, sex, country of birth, IRSD decile scores, marital status, language, Aboriginal status, employment, living arrangement, health conditions, and Crow distance
IRSD Decile	Linear regression	Treatment goal, exercise physiology, Speech Pathology, age, sex, country of birth marital status, language, Aboriginal status, employment, living arrangement, health conditions, and Crow distance
Crow distance	Linear regression	Treatment goal, exercise physiology, Speech Pathology, age, sex, country of birth, IRSD decile scores, marital status, language, Aboriginal status, employment, living arrangement, health conditions
Employment status	Multinomial logistic regression	Treatment goal, exercise physiology, Speech Pathology, age, sex, country of birth, IRSD decile scores, marital status, language, Aboriginal status, living arrangement, health conditions, and Crow distance
Health conditions	Multinomial logistic regression	Treatment goal, exercise physiology, Speech Pathology, age, sex, country of birth, IRSD decile scores, marital status, language, Aboriginal status, living arrangement, employment status, and Crow distance

Table S19 Results from imputation (outcome: treatment goals)

Imputed variables	Complete	Incomplete	Imputed	Total
Treatment goal	660	229	229	889
IRSD Decile	871	18	18	889
Crow distance	867	22	22	889
Employment status	748	141	141	889
Health conditions	736	153	153	889
